# NTRK1 knockdown induces mouse cognitive impairment and hippocampal neuronal damage through mitophagy suppression via inactivating the AMPK/ULK1/FUNDC1 pathway

**DOI:** 10.1038/s41420-023-01685-7

**Published:** 2023-10-31

**Authors:** Kai Yang, Jue Wu, Shang Li, Shan Wang, Jing Zhang, Yi-peng Wang, You-sheng Yan, Hua-ying Hu, Ming-fang Xiong, Chao-bo Bai, Yong-qing Sun, Wen-qi Chen, Yang Zeng, Jun-liang Yuan, Cheng-hong Yin

**Affiliations:** 1 Prenatal Diagnosis Center, Beijing Obstetrics and Gynecology Hospital; Beijing Maternal and Child Health Care Hospital, Capital Medical University, Beijing, 100026 China; 2 Medical Innovation Research Division, Chinese PLA General Hospital, Beijing, 100853 China; 3 Department of Anesthesiology, Peking University People’s Hospital, Beijing, 100044 China; 4 Prenatal Diagnosis Center, Shijiazhuang Obstetrics and Gynecology Hospital, Key Laboratory of Maternal and Fetal Medicine of Hebei Province, Shijiazhuang, Hebei, 050011 China; 5 Institute of Hematology, Fifth Medical Center of Chinese PLA General Hospital, Beijing, 100071 China; 6 Department of Neurology, Peking University Sixth Hospital, Peking University Institute of Mental Health, NHC Key Laboratory of Mental Health (Peking University), National Clinical Research Center for Mental Disorders (Peking University Sixth Hospital), Beijing, 100191 China

**Keywords:** Cellular neuroscience, Mitophagy

## Abstract

Hippocampal neuronal damage may induce cognitive impairment. Neurotrophic tyrosine kinase receptor 1 (NTRK1) reportedly regulates neuronal damage, although the underlying mechanism remains unclear. The present study aimed to investigate the role of NTRK1 in mouse hippocampal neuronal damage and the specific mechanism. A mouse NTRK1-knockdown model was established and subjected to pre-treatment with BAY-3827, followed by a behavioral test, Nissl staining, and NeuN immunofluorescence (IF) staining to evaluate the cognitive impairment and hippocampal neuronal damage. Next, an in vitro analysis was conducted using the CCK-8 assay, TUNEL assay, NeuN IF staining, DCFH-DA staining, JC-1 staining, ATP content test, mRFP-eGFP-LC3 assay, and LC3-II IF staining to elucidate the effect of NTRK1 on mouse hippocampal neuronal activity, apoptosis, damage, mitochondrial function, and autophagy. Subsequently, rescue experiments were performed by subjecting the NTRK1-knockdown neurons to pre-treatment with O304 and Rapamycin. The AMPK/ULK1/FUNDC1 pathway activity and mitophagy were detected using western blotting (WB) analysis. Resultantly, in vivo analysis revealed that NTRK1 knockdown induced mouse cognitive impairment and hippocampal tissue damage, in addition to inactivating the AMPK/ULK1/FUNDC1 pathway activity and mitophagy in the hippocampal tissues of mice. The treatment with BAY-3827 exacerbated the mouse depressive-like behavior induced by NTRK1 knockdown. The results of in vitro analysis indicated that NTRK1 knockdown attenuated viability, NeuN expression, ATP production, mitochondrial membrane potential, and mitophagy, while enhancing apoptosis and ROS production in mouse hippocampal neurons. Conversely, pre-treatment with O304 and rapamycin abrogated the suppression of mitophagy and the promotion of neuronal damage induced upon NTRK1 silencing. Conclusively, NTRK1 knockdown induces mouse hippocampal neuronal damage through the suppression of mitophagy via inactivating the AMPK/ULK1/FUNDC1 pathway. This finding would provide insight leading to the development of novel strategies for the treatment of cognitive impairment induced due to hippocampal neuronal damage.

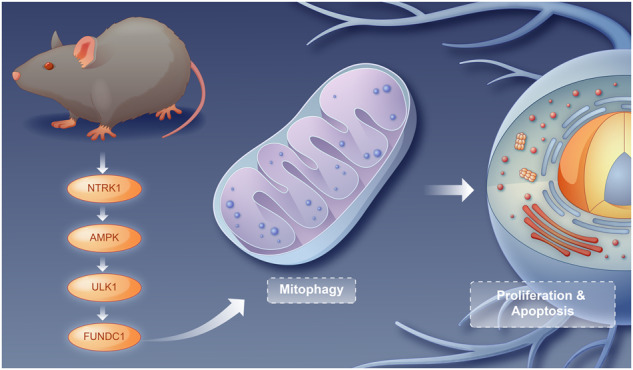

## Introduction

Cognitive impairment (CI) is induced mainly by hippocampal neuronal damage (HND), observed often in disorders such as traumatic brain injury, Alzheimer’s disease, depression, etc. [1–4]. CIhas a serious impact on the life quality of the affected patients. In recent decades, the mitigation of HND has become a research hotspot in the treatment of disorders associated with CI [5, 6]. Therefore, it is important to elucidate the mechanisms of HND so as to facilitate the development of novel strategies for the treatment of CI-associated disorders.

Neurotrophic tyrosine kinase receptor 1 (*NTRK1*, OMIM *191315; coding tyrosine kinase receptor A [TrkA]) is located on chromosome 1q23.1 and is responsible for a rare genetic disorder, CIPA (congenital insensitivity to pain with anhidrosis) [7]. In previous studies, we extended the *NTRK1* mutation spectrum and explored potential mechanism underlying CIPA [8, 9]. As a neurotrophin receptor, NTRK1 is abundantly expressed in human neuronal tissues and plays an integral role in the development and function of human nervous system [10]. NTRK1 is also involved in the regulation of the survival and proliferation of cholinergic neurons [11]. The ectopic expression of NTRK1 under the stimulation of a nerve growth factor induces the differentiation of neural stem cells to cholinergic neurons [12]. A recent study reported that an NTRK1mutation induced depression-like behavior and neuronal growth defects in mice [13]. However, the influence of NTRK1 on the biological function of hippocampal neurons remains poorly understood. In our preliminary study,NTRK1 knockdown induced apoptosis and suppressed the viability of mouse hippocampal neurons in vitro. Therefore, it was hypothesized that NTRK1 is involved in the regulation of hippocampal neuronal damage and the associated cognitive impairment. Herein, a series of systematic in vivo and in vitro experiments were conducted to investigate this hypothesis.

Mitochondria are responsible for cellular energy metabolism and regulate cell survival and death through redox control [14–16]. In neuronal cells, mitochondria are involved in the regulation of neuronal death, survival, neurotransmitter synthesis, and synaptic plasticity [17]. Mitochondrial dysfunction could induce the excessive production of free radicals, which then leads to neuronal dysfunction and, ultimately, CI [18]. Therefore, mitochondrial homeostasis is crucial for cell survival.

The maintenance of neuronal homeostasis relies on autophagy, which is the process of eliminating damaged organelles and proteins [19]. Autophagy dysfunction is one of the major pathological hallmarks of neurodegenerative disease-associated CI [20]. Interestingly, studies demonstrated that enhanced mitophagy in damaged hippocampal neurons is conducive to improving cognitive dysfunction in rats [21]. In this context, the effect of NTRK1 on mitophagy in neurons has not been elucidated. Our preliminary study revealed that mitophagy was suppressed upon NTRK1 knockdown in mouse hippocampal neurons. Based on this, this study aimed to investigate whetherNTRK1regulate mouse HND via mitophagy. A previous study suggested that the mitophagy was increased upon the activation of the adenosine monophosphate-activated protein kinase (AMPK)/Unc-51-like autophagy activating kinase 1 (ULK1)/FUN14 domain containing 1 (FUNDC1) pathway [22]. Additionally, the above-stated preliminary study indicated that NTRK1 knockdown inactivated the AMPK/ULK1/FUNDC1 pathway in mouse hippocampal neurons.

Accordingly, whether NTRK1 regulated mouse CI and HND via the AMPK/ULK1/FUNDC1 pathway-mediated mitophagy was investigated in the present study. The objective was to obtain novel insights for developing effective strategies for the treatment of CI induced by HND.

## Results

### NTRK1 knockdown in the hippocampus induced cognitive impairment in mice

In this study, the expression of NTRK1 in mouse hippocampus was regulated using a brain stereotactic injection of lentivirus carrying the NTRK1 shRNA or shRNA NC. To avoid the off-target effect,NTRK1 shRNA-1, -2 and -3 were respectively utilized to transfect mice. Two weeks after injection, the hippocampus tissues of mice were retrieved to determine the expression of NTRK1 using WB. As a result, all of the three kinds of NTRK1 shRNAs could effectively reduce the expression of mRNA and protein for NTRK1, withNTRK1 shRNA-1 showing the highest transfection efficiency (Fig. S1A–C). Hence, NTRK1 shRNA-1 was used to transfect mice in the following experiment (named the sh-NTRK1 group).

**Fig. 1:**
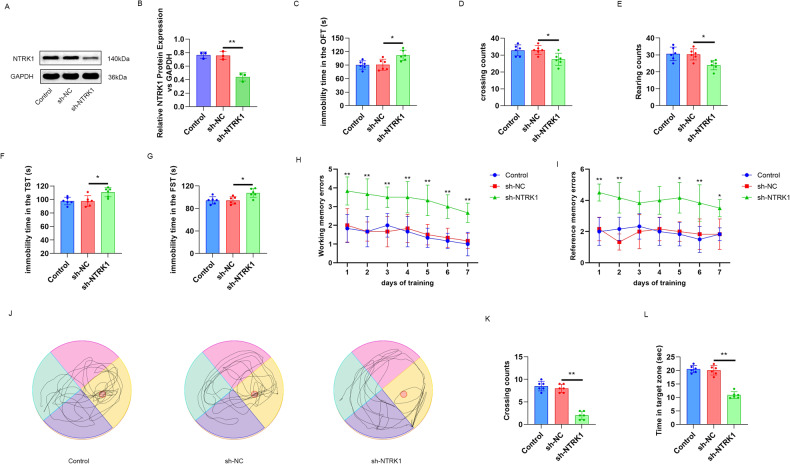
NTRK1 knockdown in mouse hippocampus induced cognitive impairment in the mice. **A**, **B** Mice were administered with a stereotactic brain injection of the lentivirus carrying NTRK1 shRNA or shRNA NC in the hippocampus. The WB results suggested that NTRK1 was effectively downregulated upon the lentiviral injection. *n* = 3. **C**–**E** The OFT test results indicated that NTRK1 knockdown increased the immobility duration and reduced the crossing and rearing counts of mice. *n* = 6. **F**, **G** The results of FST test and TST test indicated that NTRK1 knockdown prolonged the immobility duration of the mice. *n* = 6. **H**, **I** Virtual radial arm maze test suggested that NTRK1 knockdown induced the working and reference memory errors of the mice. *n* = 6. **J**–**L** Morris water maze test revealed that NTRK1 knockdown damaged the spatial memory ability of the mice. *n* = 6. *P < 0.05. **P < 0.01.

The mice of the sh-NTRK1 group exhibited a much reduced expression of the NTRK1 protein compared to the sh-NC group (*P* < 0.01) (Fig. 1A, B). In addition, the NTRK1-knockdown mice exhibited the increased immobility duration (*P* < 0.05) in the behavioral test, the reduced crossing and rearing counts (*P* < 0.05) in the OFT test (Fig. 1C–E), and the prolonged immobility duration in the FST test and TST test (*P* < 0.05) (Fig. 1F, G). Virtual radial arm maze test was performed on the mice to research the effect of NTRK1 on the working and reference memory errors. A significant impairment in the working and reference memory errors was discovered in theNTRK1-knockdown mice, as compared to the mice in the sh-NC group (*P* < 0.05 and *P* < 0.01) (Fig. 1H, I). The spatial memory ability of the mice was appraised by Morris water maze test. TheNTRK1-knockdown mice had less crossing counts and shorter time in the target zone, in comparison to the mice in the sh-NC group (*P* < 0.01) (Fig. 1J–L). All of these data indicated that NTRK1 knockdown induced mouse CI.

### NTRK1 knockdown in the hippocampus induced neuronal damage, suppressed mitophagy, and inactivated the AMPK/ULK1/FUNDC1 pathway in mice

**Fig. 2:**
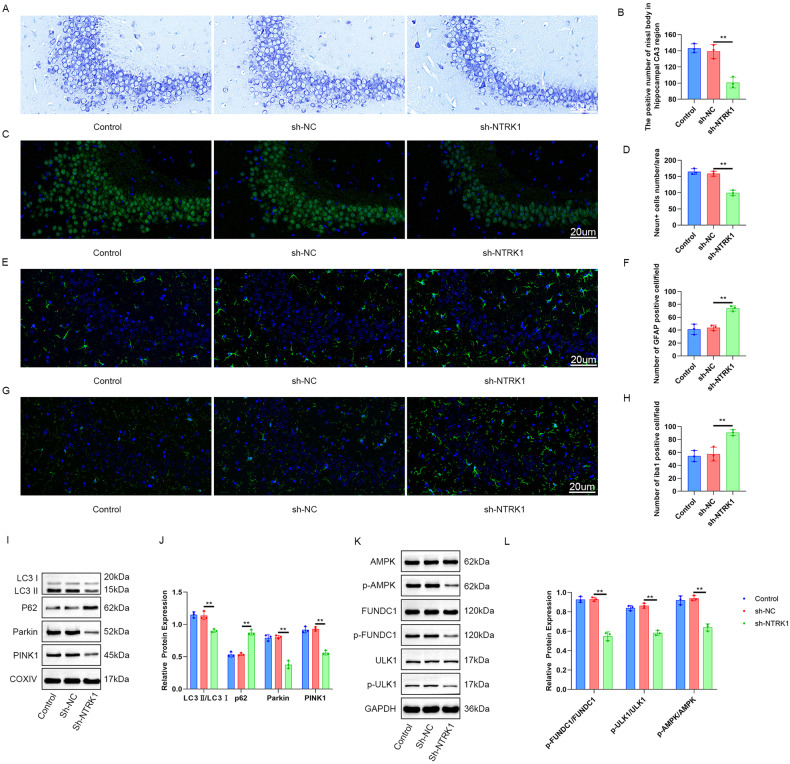
NTRK1 knockdown in mouse hippocampus tissue induced neuronal damage, suppressed mitophagy, and inactivated the AMPK/ULK1/FUNDC1 pathway in the mice. **A**, **B** Nissl staining revealed thatNTRK1 knockdown reduced the number of Nissl bodies in the mouse hippocampal CA3 region. *n* = 3. **C**–**H** The results of NeuN, GFAP, and Iba1 IF staining suggested that NTRK1 knockdown reduced the number of NeuN-positive neurons while increasing the number of GFAP and Iba1-positive neurons in mouse hippocampus. *n* = 3. **I**–**L** WB results indicated thatNTRK1 knockdown inactivated the AMPK/ULK1/FUNDC1 pathway and suppressed mitophagy in mouse hippocampus. *n* = 3. **P < 0.01.

Nissl staining of the mouse hippocampus samples revealed the effect of NTRK1 on neuronal damage. A pronounced reduction (*P* < 0.01) in the positive number of Nissl bodies was observed in the hippocampal CA3 region of the NTRK1-knockdown mice (the sh-NTRK1 group) compared to the sh-NC group (Fig. 2A, B). The expressions of NeuN, GFAP, and Iba1 in the mouse hippocampus were determined using IF staining. The mice in the sh-NTRK1 group exhibited fewer NeuN-positive neurons and greater GFAP-, Iba1-positive neurons compared to the sh-NC group (*P* < 0.01) (Fig. 2C–H). These data indicated that NTRK1 knockdown induced neuronal damage in mouse hippocampus.

In this part, mitochondria were isolated from the mouse hippocampus to investigate mitophagy. The expression of autophagy-related proteins was assessed using WB. Results revealed that the sh-NTRK1 group exhibited increased levels of P62 protein and decreased levels of LC3 II/I, Parkin, and PINK1 proteins compared to the sh-NC group (*P* < 0.01) (Fig. 2I, J). Moreover, the activity of the AMPK/ULK1/FUNDC1 pathway in the mouse hippocampus was also examined through WB. NTRK1 knockdown (sh-NTRK1 group) significantly decreased the expression of p-AMPK/AMPK, p-ULK1/ULK1, and p-FUNDC1/FUNDC1 proteins in the mouse hippocampus compared to the sh-NC group (*P* < 0.01) (Fig. 2K, L). These findings suggest that NTRK1 knockdown inhibits the AMPK/ULK1/FUNDC1 pathway, leading to the suppression of mitophagy in the mouse hippocampus.

### NTRK1 knockdown inhibited the viability and promoted the apoptosis of mouse hippocampal neurons

**Fig. 3:**
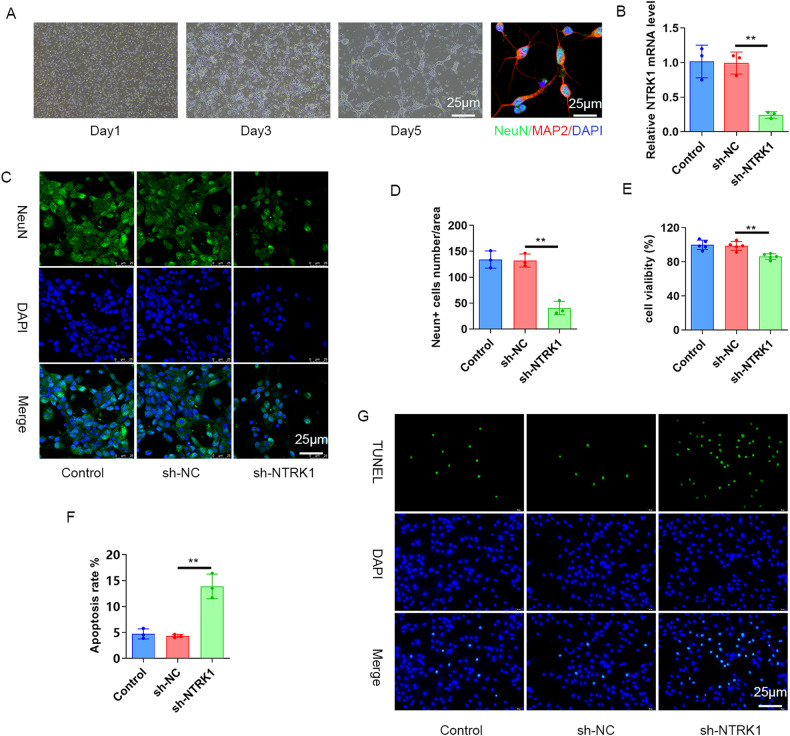
NTRK1 knockdown inhibited viability and promoted the apoptosis of mouse hippocampal neurons. **A** Mouse hippocampal neurons were isolated, cultured, and detected using NeuN/MAP2 IF staining. **B** The qRT-PCR results suggested that NTRK1 in mouse hippocampal neurons was successfully downregulated upon transfection with the NTRK1 shRNA. *n* = 3. **C**, **D** The NeuN IF staining results indicated that NTRK1 silencing reduced the number of NeuN-positive mouse hippocampal neurons. *n* = 3. **E** The CCK-8 assay revealed that NTRK1 silencing attenuated the viability of mouse hippocampal neurons. n = 6. **F**, **G** TUNEL staining indicated that NTRK1 silencing intensified the apoptosis of mouse hippocampal neurons. *n* = 3. **P < 0.01.

The role of NTRK1 in mouse hippocampal neurons was explored next. The mouse hippocampal neurons were isolated and then transfected with NTRK1 shRNA (the sh-NTRK1 group) or shRNA NC (the sh-NC group). Afterward, NeuN/MAP2 IF staining was performed, which revealed the expressions of MAP2 and NeuN in the isolated neurons, suggesting the successful isolation of mouse hippocampal neurons (Fig. 3A). The subsequent qRT-PCR analysis revealed a decrease in the NTRK1 mRNA levels in the NTRK1 shRNA-transfected mouse hippocampal neurons (the sh-NTRK1 group)compared to that in the sh-NC group (*P* < 0.01) (Fig. 3B). This suggested that NTRK1 was successfully downregulated upon sh-NTRK1 transfection into the neurons.

Furthermore, the NeuN IF staining revealed a sharp reduction in the number of NeuN-positive neurons after NTRK1 silencing (*P* < 0.01) (Fig. 3C, D). The CCK-8 assay (Fig. 3E) and TUNEL staining (Fig. 3F, G) results revealed that NTRK1 silencing resulted in decreased neuron viability and increased apoptosis of the neurons (*P* < 0.01). These data indicated that NTRK1 silencing could suppress the viability and enhance the apoptosis of mouse hippocampal neurons.

### NTRK1 knockdown disrupted the mitochondrial function and inhibited autophagy in mouse neurons

**Fig. 4:**
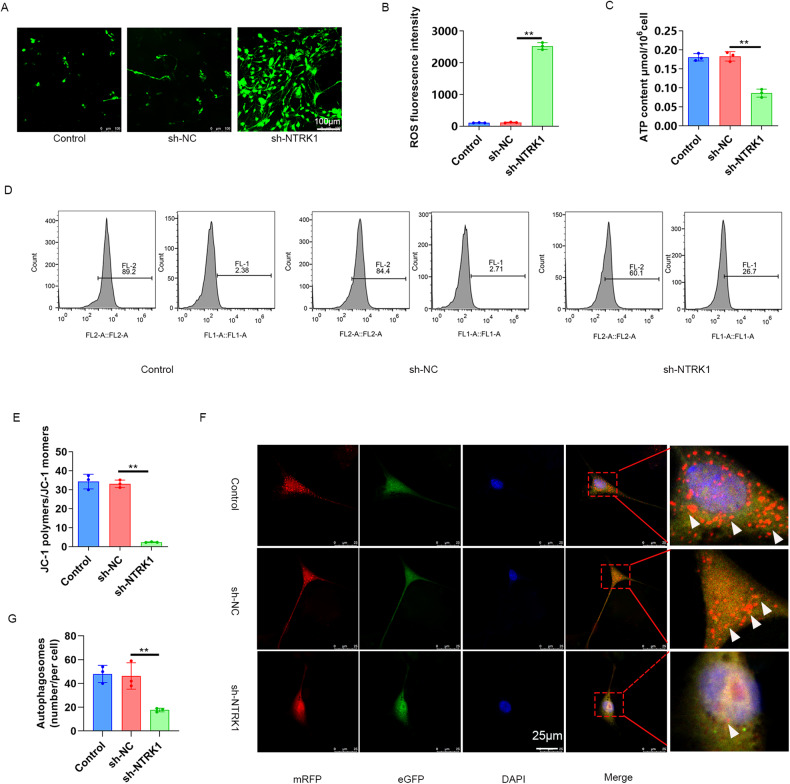
NTRK1 knockdown disrupted the mitochondrial function in mouse neurons. **A**, **B** DCFH-DA staining results suggestedthatNTRK1 knockdown enhanced ROS accumulation in mouse neurons. *n* = 3. **C** The ATP content analysis indicated that NTRK1 knockdown resulted in reduced ATP content in mouse neurons. *n* = 3. **D**, **E** The results of JC-1 staining indicated thatNTRK1 knockdown decreased the mitochondrial membrane potential in mouse neurons. *n* = 3. **F**, **G** The mRFP-eGFP-LC3 assay revealed that NTRK1 knockdown suppressed the autophagic flux in mouse neurons. *n* = 3. **P < 0.01.

The effect of NTRK1 on mitochondrial function in mouse neurons was evaluated through the evaluation of ROS accumulation, ATP content, and mitochondrial membrane potential by performing DCFH-DA staining, ATP content determination, and JC-1 staining, respectively, and the corresponding results are presented in Fig. 4A–E. In contrast to the sh-NC group, the mouse neurons from sh-NTRK1 group exhibited lower ATP content and mitochondrial membrane potential (*P* < 0.01) and generation of higher ROS. These data indicated that the NTRK1 knockdown disrupted the mitochondrial function in mouse neurons.

Subsequently, the autophagic flux in the mouse neurons was determined using the mRFP-eGFP-LC3 assay, and the results are presented in Fig. 4F, G. The mouse neurons of thesh-NTRK1 group exhibited a sharp decrease in autophagosomes compared to the sh-NC group (*P* < 0.01). Therefore,it was inferred that NTRK1 knockdown blocked the autophagic flux in mouse neurons.

### NTRK1 knockdown suppressed mitophagy in mouse neurons

**Fig. 5:**
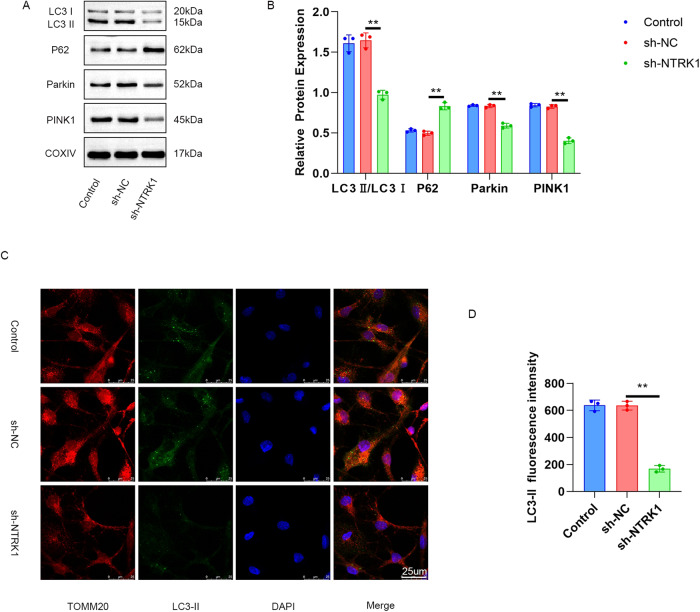
NTRK1 knockdown suppressed mitophagy in mouse neurons. **A**, **B** Mitochondria were extracted from mouse neurons. WB was performed, and it was revealed that NTRK1 knockdown suppressed mitophagy in mouse neurons. *n* = 3. **C**, **D** The results of TOMM20/LC3-II fluorescence staining indicated that silencing NTRK1 reduced the expression of Parkin1 in the mitochondria of mouse neurons. *n* = 3. **P < 0.01.

The mitochondria from mouse neurons were extracted and investigated for the expressions of autophagy-related proteins using WB. The results revealed an upregulation in the level of P62 protein and a reduction in the levels of LC3 I/II, Parkin, and PINK1 proteins in the mitochondria from thesh-NTRK1 group compared to those in the sh-NC group (*P* < 0.01) (Fig. 5A, B). In addition, the mouse neurons were subjected to TOMM20/LC3-II double fluorescence staining. While TOMM20 fluorescence staining leads to red fluorescence and indicates the respective locations of the mitochondria, LC3-II staining leads to green fluorescence. The staining results are presented in Fig. 5C, D. Mitigated LC3-II fluorescence intensity was observed in mitochondria from the sh-NTRK1 group compared to those from the sh-NC group (*P* < 0.01). Therefore, it was inferred that NTRK1 knockdown suppressed mitophagy in mouse neurons.

### NTRK1 knockdown could suppress mitophagy in mouse neurons through the inactivation of the AMPK/ULK1/FUNDC1 pathway

**Fig. 6:**
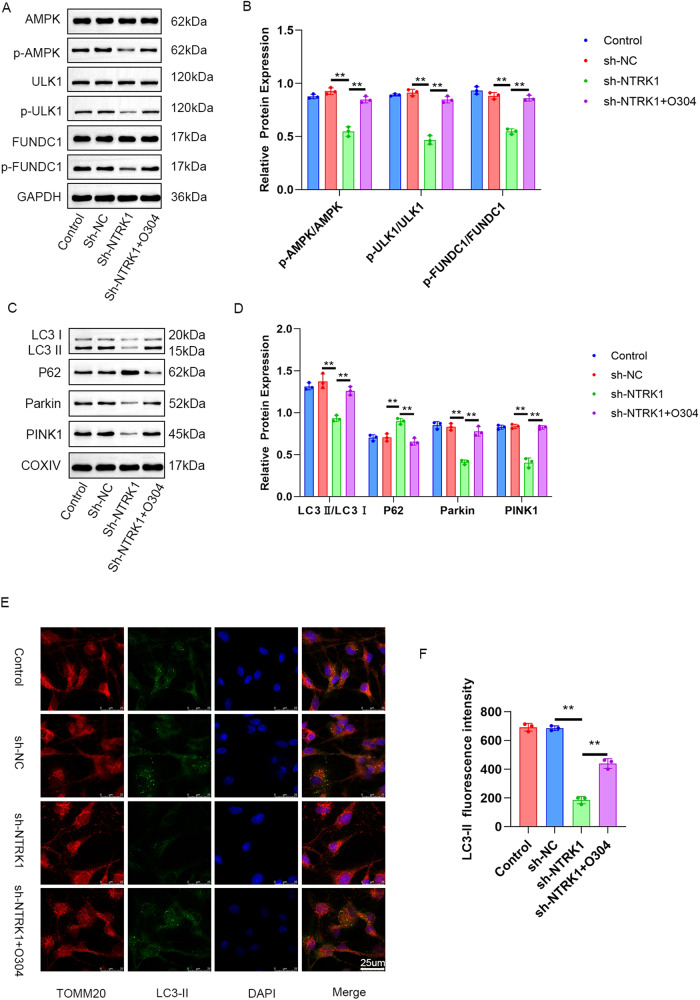
NTRK1 silencing might suppress mitophagy in mouse neurons through the inactivation of the AMPK/ULK1/FUNDC1 pathway. **A**, **B** WB revealed that O304 treatment abrogated the suppression effect of NTRK1 knockdown on the activity of the AMPK/ULK1/FUNDC1 pathway in mouse neurons. *n* = 3. **C**, **D** WB also indicated that O304 treatment counteracted the suppression effect of NTRK1 silencing on mitophagy in mouse neurons. *n* = 3. **E**, **F** The results of TOMM20/LC3-II fluorescence staining revealed that the O304 treatment reversed the suppression effect of NTRK1 knockdown on the expression of Parkin in mouse neuronmitochondria. *n* = 3. **P < 0.01.

To elucidate whether NTRK1 regulated mitophagy in mouse neurons through the activation of AMPK/ULK1/FUNDC1 pathway, the NTRK1-knockdown mouse neurons were subjected to a pre-treatment with O304, an AMPK activator. WB results revealed that the mouse neurons from the sh-NTRK1 group exhibited decreased expressions of p-AMPK/AMPK, p-ULK1/ULK1, and p-FUNDC1/FUNDC1 proteins compared to the sh-NC group (*P* < 0.01). Furthermore, an increment in the expression of p-AMPK/AMPK, p-ULK1/ULK1, and p-FUNDC1/FUNDC1 proteins was observed in the mouse neurons from the sh-NTRK1 + O304 group compared to the sh-NTRK1 group (*P* < 0.01) (Fig. 6A, B). These results confirmed that pre-treatment with O304 abrogated the suppression effect of NTRK1 knockdown on the activity of the AMPK/ULK1/FUNDC1 pathway in mouse neurons.

Next, mitochondria were extracted from the mouse neurons of each group and evaluated for the expressions of autophagy-related proteins by WB. The results revealed that compared to the sh-NC group mitochondria, those from thesh-NTRK1 group exhibited a lower expression of LC3 II/I, Parkin, and PINK1 proteins and higher levels of the P62 protein(*P* < 0.01). Conversely, an increment in the expressions of LC3 II/I, Parkin, and PINK1 proteins, as well as a reduction in the expression of P62 protein, was observed in the mitochondria from thesh-NTRK1 + O304 group, compared to the sh-NTRK1 group(*P* < 0.01) (Fig. 6C, D). The subsequent TOMM20/LC3-II fluorescence staining revealed a weakened LC3-II fluorescence intensity in the mitochondria of the sh-NTRK1 group compared to the sh-NC group(*P* < 0.01). Conversely, intensified LC3-II fluorescence staining was detected in the mitochondria from the sh-NTRK1 + O304 group (*P* < 0.01) in contrast to the sh-NTRK1 group (Fig. 6E, F). These results indicated that treatment with O304 counteracted the suppression effect of NTRK1 knockdown on mitophagy in mouse neurons.

Besides, to investigate whether NTRK1 knockdown mediated mitophagy in mouse neurons by regulating Parkin, PINK1 or FUNDC1, this study implemented rescue experiment on mouse neurons. The results were presented in Fig. S2A–C. TUNEL assay and CCK-8 assay showed that, NTRK1 knockdown markedly induced the apoptosis but attenuated the viability of mouse neurons (sh-NTRK1 group vs. sh-NC group; *P* < 0.01). However, these effects of NTRK1 knockdown on the apoptosis and viability of mouse neurons was reversed by the overexpression of FUNDC1 (sh-NTRK1 + OE FUNDC1 group vs. sh-NTRK1 group; *P* < 0.05 and *P* < 0.01). The difference in the apoptosis and viability of mouse neurons was similar among the sh-NTRK1 group, the sh-NTRK1 + OE Parkin group and the sh-NTRK1 + OE PINK1 group. Thus, FUNDC1 overexpression (rather than Parkin and PINK1) abrogated the neuronal damage induced by NTRK1 knockdown.

Collectively, all of these above results demonstrated that the inhibition of mitophagy in mouse neurons upon NTRK1 knockdown might involve the inactivation of the AMPK/ULK1/FUNDC1 pathway.

### NTRK1 knockdown could induce neuronal damage through the inhibition of mitophagy

**Fig. 7:**
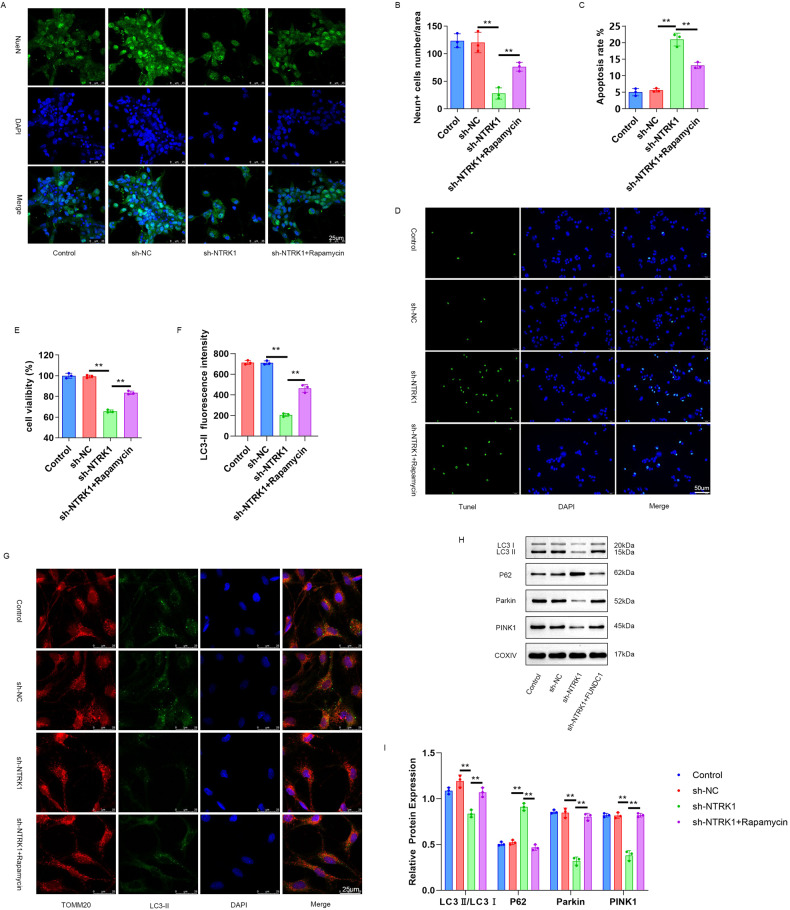
NTRK1 silencing might induce neuronal damage through the suppression of mitophagy. **A**, **B** The results of NeuN IF staining revealed that rapamycin treatment counteracted the suppression effect of NTRK1 silencing on the expression of NeuN in mouse neurons. *n* = 3. **C**, **D** TUNEL staining indicated that rapamycin treatment reversed the promotion effect of NTRK1 silencing on the apoptosis of mouse neurons. *n* = 3. **E** The CCK-8 assay revealed that rapamycin treatment reversed the inhibition effect of silencing NTRK1 on the viability of mouse neurons. *n* = 3. **F**, **G** TOMM20/LC3-II fluorescence staining results revealed that rapamycin treatment abrogated the inhibition effect of NTRK1 silencing on the expression of Parkin1 in the mitochondria of mouse neurons. *n* = 3. **H**, **I** Mitochondria were extracted from the mouse neurons of each group. WB was performed, and it was revealed that rapamycin treatment reversed the suppression effect of NTRK1 silencing on mitophagy. *n* = 3. **P < 0.01.

To verify whether NTRK1 regulated the mouse neuronal function through the regulation of mitophagy, the NTRK1-knockdown mouse neurons were pre-treated with rapamycin, an autophagy activator. The subsequent NeuNIF staining and TUNEL staining results revealed a reduced number of NeuN-positive neurons and an increased neuronal apoptosis rate in the sh-NTRK1 group compared to the sh-NC group (*P* < 0.01). On the contrary, the mouse neurons from the sh-NTRK1 + Rapamycin group presented a higher number of NeuN-positive neurons and a lower neuronal apoptosis rate compared to the sh-NTRK1 group (*P* < 0.01) (Fig. 7A–D). Furthermore, the CCK-8 assay results revealed a reduction in neuronal viability in the sh-NTRK1 group compared to the sh-NC group (*P* < 0.01). However, the mouse neurons from the sh-NTRK1 + Rapamycin group exhibited enhanced viability compared to the sh-NTRK1 group (*P* < 0.01) (Fig. 7E). Moreover, attenuated LC3-II fluorescence staining intensity was observed in the mitochondria from the sh-NTRK1 group compared to the sh-NC group (*P* < 0.01). On the contrary, compared to the sh-NTRK1 group mitochondria, the mitochondria from the sh-NTRK1 + Rapamycin group exhibited increased LC3-II fluorescence staining intensity (*P* < 0.01) (Fig. 7F, G).

Meanwhile, mitochondria from each were also subjected to WB. The results revealed that NTRK1 knockdown evidently decreased the expressions of LC3 II/I, Parkin, and PINK1 proteins while elevating the expression of P62 protein in these mitochondria, comparatively (sh-NTRK1 group vs. sh-NC group; *P* < 0.01). However, rapamycin treatment abrogated these effects of NTRK1 knockdown on the expressions of the above-stated proteins (sh-NTRK1 + rapamysin group vs. sh-NTRK1 group; *P* < 0.01) (Fig. 7H, I). Collectively, these results indicated that NTRK1 knockdown might disrupt mouse neuronal function through the suppressing of mitophagy.

### NTRK1knockdown might induce CI in mice through the inactivation of the AMPK/ULK1/FUNDC1 pathway

**Fig. 8:**
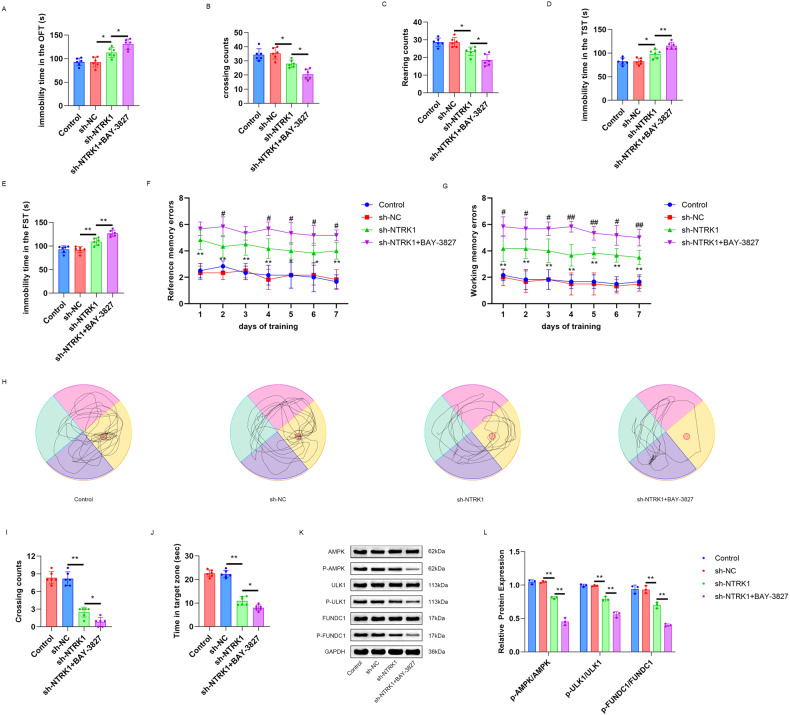
NTRK1 knockdown might induce mouse cognitive impairment through the inactivation of the AMPK/ULK1/FUNDC1 pathway. **A**–**C** The OFT test revealed that BAY-3827 treatment further prolonged the immobility duration and reduced the rearing and crossing counts of the mice with NTRK1 knockdown. *n* = 6. **D**, **E** The results of TST test and FST test suggested that BAY-3827 treatment further prolonged the immobility duration of the mice with NTRK1 knockdown. n = 6. **F**, **G** Virtual radial arm maze test suggested that BAY-3827 treatment intensified the working and reference memory damage in themice with NTRK1 knockdown. *n* = 6. **H**–**J** Morris water maze test demonstrated that BAY-3827 treatment enhanced the damaged of the spatial memory ability in the mice with NTRK1 knockdown. *n* = 6. **K**, **L** WB demonstrated that BAY-3827 treatment further decreased the AMPK/ULK1/FUNDC1 pathway activity in the hippocampus of the mice with NTRK1 knockdown. *n* = 3. *P < 0.05. **P < 0.01.

To explore whetherNTRK1 regulated CI in mice via the AMPK/ULK1/FUNDC1 pathway, the NTRK1-knockdown mice were treated withBAY-3827, a selective inhibitor of AMPK, and the results are displayed in Fig. 8A–C. NTRK1 knockdown prolonged the immobility duration (*P* < 0.05) and reduced the rearing and crossing counts (sh-NTRK1 group vs. sh-NC group; *P* < 0.05) of mice in the OFT test. Interestingly, this effect of NTRK1 knockdown was further enhanced upon treatment with BAY-3827(sh-NTRK1 + BAY-3827 group vs. sh-NTRK1 group; *P* < 0.05). Moreover, in the FST test and the TST test, NTRK1 silencing prolonged the immobility duration in the mice (sh-NTRK1 group vs. sh-NC group; *P* < 0.05 or *P* < 0.01, respectively),which was further prolonged upon treatment with BAY-3827(sh-NTRK1 + BAY-3827 group vs. sh-NTRK1 group; *P* < 0.01) (Fig. 8D, E). Besides,NTRK1knockdownenhancedthe working and the reference memory errors of mice in the Virtual radial arm maze test (sh-NTRK1 group vs. sh-NC group; *P* < 0.05 and *P* < 0.01), but reduced the crossing counts and the time in the target zone (sh-NTRK1 group vs. sh-NC group; *P* < 0.01). These phenomena were further enhanced by BAY-3827 treatment (sh-NTRK1 + BAY-3827 group vs. sh-NTRK1 group; *P* < 0.05 and *P* < 0.01) (Fig. 8F–J).

The results of the subsequent WB analysis of the mouse hippocampus tissue revealed that NTRK1 knockdown suppressed the expressions of p-AMPK/AMPK, p-ULK1/ULK1, and p-FUNDC1/FUNDC1 proteins in the mouse hippocampus(sh-NTRK1 group vs. sh-NC group; *P* < 0.01). Intriguingly, BAY-3827 treatment further decreased the expressions of the above proteins(sh-NTRK1 + BAY-3827 group vs. sh-NTRK1 group; *P* < 0.01) (Fig. 8K–L). Collectively, these data indicated thatNTRK1 knockdown might induce CI in mice through the inactivation of the AMPK/ULK1/FUNDC1 pathway.

## Discussion

NTRK1 has been reported to contribute to neuronal survival, differentiation, and development [23, 24], which encouraged the present investigation of whetherNTRK1was involved in the regulation of the CI due to HND. Therefore, a series of systematic experiments were conducted in this study, which revealed thatNTRK1 knockdown induced mouse CI and mouse HND. Mechanistically,NTRK1 knockdown damaged mouse hippocampal neurons through the suppression of mitophagy via inactivating the AMPK/ULK1/FUNDC1 pathway. In other words, NTRK1 exerted a protective effect on the hippocampal neurons. This finding may provide insight into the development of novel strategies for CI treatment.

In our study, NTRK1 was successfully silenced in mice by a stereotaxic brain injection of lentivirus, and the induction of CI in these mice was revealed through behavioral tests. Hippocampus is recognized as an important region in brain which is associated with cognition, and CI is reported to be induced mainly through HND [25–28]. Nissl body assessment is frequently used in the evaluation of neuronal viability, as the number of Nissl bodies is greatly reduced in damaged neurons [29]. Furthermore, NeuN is a key neuron-specific nuclear protein that is used as a marker of mature neurons, and the absence of its expression implies attenuated neuronal viability [30, 31]. Herein, NTRK1 knockdown resulted in a decrease in the number of Nissl bodies and NeuN-positive neurons in mouse hippocampal tissues. Conversely, elevated expressions of GFAP and Iba1 were observed in the mouse hippocampal tissues upon NTRK1 knockdown. GFAP and Iba1 are two kinds of neuroinflammatory biomarkers used in the assessment of neuronal damage and neurological deficits [32]. The neuroinflammation induced by GFAP and Iba1is reportedly associated with CI [33]. Therefore, NTRK1 knockdown could induce a neuroinflammatory response by enhancing the expressions of GFAP and Iba1, thereby inducing damage to the mouse hippocampal tissues. In order to further confirm the damage caused by NTRK1 knockdown, mouse hippocampal neurons expressing the neuronal markers NeuN and MAP2 [34] were successfully isolated. IF results (Fig. 3) indicated that NTRK1 knockdown weakened the viability and enhanced the apoptosis of the isolated mouse hippocampal neurons. Previous studies indicated the neuroprotective functions of NTRK1 [10, 11, 23, 24]. So consistently, our findings also indicated the neuroprotective role of NTRK1 on hippocampal neurons.

Recently, it was reported that mutatedNTRK1 led to neurodegeneration by inducing dysfunctional autophagic flux [35]. Moreover, a study revealed that the ectopic expression of NTRK1 induced nerve growth factor-dependent cell death in the tumor of the nervous system via autophagy [36]. Mitophagy is essential for the maintenance of normal cellular function since it facilitates the removal of damaged organelles or proteins that could otherwise disturb the normal functions of cell [37]. In this study, mitochondria were isolated from mouse hippocampal tissues and the autophagy-related proteins in them were evaluated. It was revealed that theP62 protein expression was upregulated while the LC3 II/I, Parkin, and PINK1 proteins were downregulated by NTRK1 knockdown. Similar results were observed when the mitochondria from isolated mouse hippocampal neurons were subjected to the same analysis. Increased P62 level generally indicates suppressed autophagy, while reduced P62 level indicates the enhancement of the autophagy flux [38]. Parkin and PINK1 are mediators in the selective degradation of damaged mitochondria through mitophagy [39]. LC3 II/I is reportedly recruited on the outer membrane of mitochondria to facilitate the formation of autophagosomes [40]. Therefore, these findings collectively indicated that NTRK1 knockdown suppressed mitophagy in mouse hippocampal neurons or tissues.

Furthermore, our study investigated the effect of NTRK1 knockdown on the mitochondrial function of the isolated mouse hippocampal neurons. The results indicated that NTRK1 knockdown increased the production of ROS while reducing the production of ATP in mouse hippocampal neurons. More importantly,NTRK1 silencing induced a decrease in the mitochondrial membrane potential and suppressedmitophagy in mouse hippocampal neurons. Neurons rely highly on the mitochondrial production of ATP to fulfill their energy requirements, and impaired mitochondrial function reportedly induces neuronal damage and consequently neurological disorders [41]. Moreover, mitochondria are the key regulators of cellular redox homeostasis, and damage to mitochondria could lead to an excessive accumulation of intracellular ROS, which could result in cell death [42]. Therefore, based on our results, it could be inferred thatNTRK1 knockdown led to disrupted mitochondrial function in mouse hippocampal neurons. Furthermore, treatment with rapamycin, an autophagy activator, reversed the NTRK1 knockdown-induced damage to mouse hippocampal neurons. Meanwhile, rapamycin abrogated the suppression of NTRK1 knockdown on the expression of LC3 II in mitochondria. This was, to the best of our knowledge, the first time that NTRK1 knockdown was demonstrated to induce mouse hippocampal neuron damage through the suppression of mitophagy.

Interestingly, our study also revealed that NTRK1 knockdown activated the AMPK/ULK1/FUNDC1 pathway both in mouse hippocampal tissues and the isolated mouse hippocampal neurons. AMPK is an energy sensor and regulator that controls mitochondrial biogenesis, morphology, and mitophagy, serving as a key regulator of mitochondrial homeostasis [17]. The phosphorylation of the components of the AMPK pathway maintains the biogenesis and normal functioning of the mitochondria [43]. ULK1 is a substrate for AMPK phosphorylation which is capable of regulating mitochondrial function via direct coupling with AMPK [44, 45]. Moreover, activated ULK1 promotes the phosphorylation of FUNDC1, a receptor protein that is located on the mitochondrial membrane [46, 47]. It has been discovered that, in cerebral ischaemia-reperfusion injury, FUNDC1could suppress the neuronal apoptosis and improve mitochondrial function [48]. Under non-hypoxic conditions, such as the diabetic CI and the epileptic neuronal injury, FUNDC1 was observed to protect neurons from damage by enhancing mitosis and neuronal viability and attenuating neuronal apoptosis [49, 50]. These previous data suggested thatFUNDC1played a neuronal protective role under both hypoxic and non-hypoxic conditions. Interestingly, in the current work, NTRK1 knockdown was suggested to inhibit the expression of p-FUNDC1/FUNDC1 (rather than FUNDC1). Besides, rescue experiment indicated that FUNDC1 overexpression (rather than Parkin and PINK1) reversed the neuronal damage induced by NTRK1 knockdown. Taken together, p-FUNDC1 level might be increased upon FUNDC1 overexpression, thereby alleviating the neuronal damage induced by NTRK1 knockdown. Thus, the novel finding of this paper was thatNTRK1 knockdown might induce the neuronal damage by suppressing the phosphorylation of FUNDC1. Previous studies have demonstrated that the activation of the components of the AMPK/ULK1/FUNDC1 pathway could play a mediatory role in mitochondrial formation and mitochondrial phagocytosis to prevent neuronal apoptosis [51, 52]. The in vitro experiments conducted in our study indicated that treatment with O304, an AMPK activator, counteracted the suppression effect of NTRK1 knockdown on mitophagy in mouse hippocampal neurons. In addition, in vivo results indicated that treatment with BAY-3827, a selective inhibitor of AMPK, further exacerbated the mouse CI induced uponNTRK1 knockdown. Therefore, it was inferred thatNTRK1 knockdown might induce mouse CI, at least partially, via suppressing mitophagy in the hippocampal neurons through the activation of the AMPK/ULK1/FUNDC1 pathway.

Conclusively, NTRK1 knockdown was indicated to impair cognitive function and hippocampal neurons in mice, and also to inactivate the AMPK/ULK1/FUNDC1 pathway and suppressmitophagy in mouse hippocampal neurons. Mechanistically,NTRK1 knockdown might induce cognitive impairment and hippocampal neuron damage through the suppression of mitophagy via inactivating theAMPK/ULK1/FUNDC1 pathway. Therefore,it is inferred that NTRK1exerts a neuroprotective effect on the hippocampus. Accordingly, NTRK1 could be a promising candidate therapeutic target in the treatment of cognitive impairment associated with hippocampal neuronal damage.

## Materials and methods

### Animals and ethical approval

C57BL/6J mice (including 27 male and 27 female, *n* = 54; 8-week-old, weighing 23–25 g) were procured from Vital River (Beijing, China). All mice were housed in a ‘non-specific pathogens’ room maintained at 22 °C with a 12-h day/12-h night cycle. All mice had free access to food and water. Animal deaths did not occur throughout the course of the experiment. No samples or animals were excluded from this study. The random number table method following the random double-blind principle was used to group the animals.

The procedures of the present study were approved by the Animal Ethics Committee of Chinese PLA General Hospital (approval no. 2018-X14–40). Animal treatment was performed in accordance with the Guide for the Care and Use of Laboratory Animals.

### Mouse transfection via brain stereotactic injection

A total of 30 mice were selected randomly and divided into the control group, the sh-NC group, the sh-NTRK1-1 group, the sh-NTRK1-2 group and the sh-NTRK1-3 group (*n* = 6 in each group). Mice in the sh-NC group and the sh-NTRK1-1, -2 and -3 groups were administered the brain stereotactic injection of lentivirus carrying the shRNA negative control (NC) or the NTRK1 shRNA-1 (sequences: 5′-TAATAGTCGGTGCTGTAGATA-3′), -2 (sequences: 5′-AAGTATTGTGGGTTCTCGATG-3′) and -3 (sequences:5′-TAGATATCCCTGCTCATGCCA-3′), respectively. These lentiviruses were constructed and provided by GenePharma(Shanghai, China). In brief, the mice were anesthetized using 5% isoflurane (w100, KeYueHuaCheng Technology, Beijing, China), and the anesthesia was subsequently maintained using 2% isoflurane. When the mice were unresponsive to the stimulation on limbs or head, they were immobilized on a brain stereotaxic apparatus (1056199-I,Zhongshi Di Chuang Technology, Beijing, China). The hair on mouse head was shaved, and the visible skin was then cut to expose the hippocampal region [2.0 mm posterior to the anterior chimney point, 1.5 mm to the left and right, and 2.0 mm in depth]. The exposed hippocampal region was drilled to insert a microsyringe, through which 1 µL (titer: 1 × 10^9^) of the lentivirus was injected into the hippocampal region at an rate of 0.2 µL/min. After injection, the microsyringe was left undisturbed in its place for 10 min and then was gradually withdrawn. The head of each mouse was then sealed with bone wax, after which the skin was sutured and disinfected. The mice in control group were subjected to the same surgical procedure without the lentivirus injection. After surgery, all mice were maintained for two weeks.

### BAY-3827 treatment of mouse

The rest 24 mice were divided randomly into the control group, the sh-NC group,the sh-NTRK1 group, and the sh-NTRK1 + BAY-3827 group (*n* = 6 in each group). The mice in the control group,the sh-NC group, and the sh-NTRK1 group were treated as described above in the “Mouse transfection through brain stereotactic injection” section.

BAY-3827,a selective inhibitor of AMPK, was purchased from Yuanxi Biotechnology (Shanghai, China) and dispersed in phosphate buffered solution (PBS) to prepare a concentration of 100 µM/L. Next, the mice in the sh-NTRK1 + BAY-3827 group were administered a brain stereotactic injection of BAY-3827 (5 µL per mouse). After 1 h, the mice were injected with the lentivirus carrying shRNA-NTRK1. All mice were then maintained for two weeks.

### Open field test (OFT), tail suspension test (TST), and forced swimming test (FST)

Behavioral tests, including OFT, TST, and FST, were conducted to investigate the effect of NTRK1 knockdown on mouse cognition.

The OFT test was performed as follows. The mice were placed in a 50 × 50 cm^2^ open field arena (with a black wall at the height of 40 cm). The open field was divided into three zones: the central circlezones (10 cm in diameter),the outer circlezones (37 cm in diameter), and the wall zone (the remaining area of the arena). Prior to the test, the mice were allowed to explore the arena freely for 60 min. Afterwards, the test was conducted for 5 min. The immobility duration, the central crossing counts, and the rearing counts of mice were recorded during this period using the ANY-maze software.

In the TST test, the mice were placed individually inside sound-isolated rooms, suspended from their tail ends, using adhesive tape at a distance of 50 cm from the floor. The mice were suspended for a total of 6 min, in which the immobility duration was recorded in the last 4 min using the ANY-maze software.

In the FST test,the mice were placed individually inside glass cylinders [14 cm in length, 14 cm in width, and 20 cm in height] containing fresh water (25 ± 2 °C) for 6 min. The depth of water in each glass cylinder was 10 cm. The immobility duration was defined as the time a mouse spent floating motionless. The immobility duration was recorded in the last 4 min using the ANY-maze software.

### Virtual radial arm maze test

This study performed the virtual radial arm maze test to investigate the working and reference memory of mice during the last week. Briefly, mice were placed in a maze with six arms. Reward pellets were placed in three of the six arms. Mice were required to find the three reward pellets, and then returned to the center of the maze for the next trial. For each mice, five trials were experienced with atrial interval of 10 s. The working memory errors was evaluated by the times of mice reentered the same arm, while the reference memory errors were assessed by the times of mice reentered the arms without reward pellets.

### Morris water maze test

To research the spatial memory ability, mice were subjected to the Morris water maze test. Mice were trained four times daily for 1–5 days prior to the trial. The training interval was 20 min. In each trial, mice were required to find the round platform (a diameter of 10 cm) in the target quadrantin a circular pool (a diameter of 180 cm). The round platform was submerged 2 cm under the water. Besides, mice were required to stay on the platform for 10 s. The training time was 2 min. After training, the Morris water maze test was started. The crossing counts and the time in the target zone were recorded under a camera with a computerized-tracking system.

### Euthanasia of the mice and retrieval of the hippocampus

After conducting the behavioral tests, the mice were euthanized. Briefly, the mice were first subjected to deep anesthesia using 5% isoflurane. When the limbs and head of mice were unresponsive to stimulation, they were rapidly euthanized by neck dislocation. After 10 min, all mice stopped breathing. The hippocampus was then retrieved from all euthanized mice and stored inside a refrigerator at −80 °C.

### Nissl staining

The mice hippocampus samples were paraffin-embedded and excised into 4-µm sections. After dewaxing in xylene, the sections were rehydrated in a gradient of alcohol solutions. Toluidine blue (C0117, Beyotime, Shanghai, China) was added to the sections, and staining was allowed for 10 min at room temperature. Afterwards, the sections were washed with distilled water to remove the residual staining solution, then were dehydrated using a gradient alcohol solution and turned transparent using xylene. After drying, the sections were sealed in neutral resin. The number of Nissl bodies in the hippocampal CA3 region was photographed under an optical microscope (CX41, Olympus, Tokyo, Japan) and quantified using the Image-Pro Plus software (Media Cybernetics, Bethesda, MD, USA).

### Isolation and culture of mouse hippocampal neurons

The newborn C57BL/6 male mouse (Vital River, Beijing, China) was euthanized to collect the hippocampal tissues. The hippocampal tissues were excised into small pieces and then digested with0.125% trypsin (T8150, Solarbio, Beijing, China) at 37 °C for 15 min. The cells were collected through centrifugation and then resuspended in the neurobasal medium (21103049, BofeiDongfang Biotechnology, Beijing, China) containing 2% B27 (17504044, Solarbio, Beijing, China) and 0.25% Glumax (35050061, Sai Cheng Biotechnology, Guangzhou, China). The cell suspension (1 × 10^6^ cells/mL) was then cultured in a 10-mm dish coated with10 mM/L of poly-d-lysine (ST5028, Beyotime, Shanghai, China). After three days of culture, 2.5 µg/mL of cytosine arabinoside (C8040, Solarbio, Beijing, China) was added to the culture dish, followed by cell culture for 24 h to suppress the proliferation of glial cells. After 24 h, 50% of the medium in the culture dish was changed and refreshed every 3 days, and the cells were cultured for 2 weeks at 37 °C in a 5% CO_2_ atmosphere followed by identification.

### Identification of mouse hippocampal neurons

Neuronal nuclei (NeuN) and microtubule-associated protein 2 (MAP2) are two key neuronal markers. In this experiment, cells isolated from mouse hippocampal tissues were identified using NeuN/MAP2 IF staining. Briefly, after 2 weeks of culture, the cells were sequentially fixed using 4% paraformaldehyde (P1110, Solarbio, Beijing, China), blocked using 5% bovine serum albumin (A8020, Solarbio, Beijing, China) for 30 min at room temperature, and then probed overnight using rabbit anti-NeuN (1:100, ab104225, Abcam, Shanghai, China) and mouse anti-MAP2 (1:100, MAB11155, AmyJet Scientific, Wuhan, China) at 4 °C. Next, the cells were treated with Alexa Fluor488-conjugated goat anti-rabbit secondary antibody (1:200, D110061, Sangon Biotech, Shanghai, China) and Alexa Fluor 594-conjugated goat anti-mouse secondary antibody (1:200, 115–585–146, AmyJet Scientific, Wuhan, China) for 2 h at room temperature. Afterward, the cells were subjected to nuclear staining using 4′,6-diamidino-2-phenylindole (DAPI)(1:30, C0065, Solarbio, Beijing, China). Finally, the expressions of NeuN (green fluorescence) andMAP2 (red fluorescence) in the cells were observed and photographed under a fluorescence microscope (BX51, Olympus, Tokyo, Japan).

### Transfection and treatment of mouse hippocampal neurons

The mouse hippocampal neurons were successfully isolated and cultured in the normal neurobasal medium at 37 °C and 5% CO_2_ atmosphere for 48 h. Next, the neurons were seeded in the wells of 6-well plates containing serum-free medium. A total of 1 × 10^6^ neurons in 1 mL of serum-free medium were seeded in each well. NTRK1 shRNA(shRNA-1) or negative NC (GenePharma, Shanghai, China) was transfected in these neurons using Lipofectamine 3000 (L3000001, Thermo Fisher Scientific, San Jose, CA USA), and the resultant transfected neurons were designated as the sh-NTRK1 group and the sh-NC group, respectively. Further, neurons were pre-treated with50 nMO304(AMPK activator)(S88333, Yuanye Biotechnology, Shanghai, China) for 1 h and then transfected with NTRK1 shRNA, and these neurons were designated as the sh-NTRK1 + O304 group. The neurons treated with 2 µM of rapamycin (autophagy activator) (B20714, Yuanye Biotechnology, Shanghai, China) and then transfected with the NTRK1 shRNA formed the sh-NTRK1 + rapamycin group. For the cotransfection, NTRK1 shRNA(shRNA-1) combined with Parkin vectors, or with PINK1 vectors or with FUNDC1 vectors were transfected into the mouse hippocampal neurons according to the instructions of Lipofectamine 3000 (named the sh-NTRK1 + OE Parkin group, the sh-NTRK1 + OE PINK1 group and the sh-NTRK1 + OE FUNDC1 group, respectively). These vectors expressing Parkin, PINK1 and FUNDC1 were purchased from GenePharma (Shanghai, China). After transfection, the neurons in each group were cultured in the normal neurobasal medium at 37 °C and 5% CO_2_ atmosphere for 48 h. The neurons, without any treatment, formed the Control group.

### Cell counting kit-8 (CCK-8) assay

The in vitro viability of mouse hippocampal neurons was evaluated using the CCK-8 assay. Briefly, mouse hippocampal neurons in each group were cultured in the wells of 96-well plates (5 × 10^3^ neurons in 100 µL of culture medium per well) at 37 °C and 5% CO_2_ atmosphere for 24 and 48 h. The neurons in each well were then treated with 10 µL of the CCK-8 solution (abs50003, Absin Biotechnology, Shanghai, China) at 37 °C for 2 h. The absorbance of each well was read at 450 nm using amicroplate reader (Biotek, Winooski, VT, USA). The viability of the neurons was calculated as follows: the absorbance of the experiment group/blank group × 100%. The absorbance of the culture medium was used as the blank value.

### Immunofluorescence (IF) staining

The expressions of NeuN, glial fibrillary acidic protein (GFAP), and ionized calcium-binding adaptor molecule 1 (Iba1) in mouse hippocampal tissues were detected using NeuN, GFAP, and Iba1 IF staining, respectively. Briefly, the hippocampal tissue sections retrieved from the mice were dewaxed, rehydrated, and then blocked with 5% bovine serum albumin (A8020, Solarbio, Beijing, China) for 30 min at room temperature. Next, the sections were probed using rabbit anti-NeuN (1:100, ab104225, Abcam, Shanghai, China), anti-GFAP (1:100, ab211271, Abcam, Shanghai, China), and anti-Iba1 (1:100, ab178846, Abcam, Shanghai, China) at 4 °C for 12 h and then incubated with Alexa Fluor488-conjugated goat anti-rabbit secondary antibody (1:200, D110061, Sangon Biotech, Shanghai, China) for 2 h at room temperature. Afterward, DAPI (1:30, C0065, Solarbio, Beijing, China) was added for nuclear staining. Subsequently, the sections were dehydrated, rendered transparent, and finally sealed in neutral resin. The IF of the NeuN, GFAP, and Iba1 staining(green fluorescence) was observed and photographed under the fluorescence microscope (BX51) and quantified using the Image-Pro Plus software (Media Cybernetics, Bethesda, MD, USA).

The expression of NeuN in the isolated mouse hippocampal neurons was evaluated using NeuNIF staining. Neurons were cultured in the wells of 6-well plates (1 × 10^6^ neurons in 1 mL of culture medium per well) under relevant conditions for 48 h. Subsequently, the neurons were immobilized using 4% paraformaldehyde (P1110, Solarbio, Beijing, China) at room temperature for 10 min and then subjected to NeuNIF staining as described above. TheNeuN-positive neurons were observed and photographed under the fluorescence microscope(BX51) and quantified using the Image-Pro Plus software (Media Cybernetics).

Further, to evaluate mitochondria autophagy, mouse hippocampal neurons in each group were stained using the primary antibodies named rabbit anti-TOMM20 (translocase of outer mitochondrial membrane 20) (1:100, ab186735, Abcam, Shanghai, China) and mouse anti-LC3II (1:100, VB2930-50, AmyJet Scientific, Wuhan, China) at 4 °C for 12 h. Afterwards, the neurons were incubated with the Alexa Fluor594-conjugated goat anti-rabbit secondary antibody (1:200, 111–585–003, AmyJet Scientific, Wuhan, China) and Alexa Fluor488-conjugated goat anti-mouse secondary antibody (1:200, 115–545–044, AmyJet Scientific, Wuhan, China) for 2 h at room temperature. TheParkin1 fluorescence intensity (green fluorescence) was observed under the fluorescence microscope(BX51) and qualified using the Image-Pro Plus software (Media Cybernetics).

### TdT-mediated dUTP nick-end labeling (TUNEL) assay

The apoptosis of hippocampal neurons was detected in vitro using the TUNEL assay commercial kit (11684817910, Roche, Shanghai, China) strictly in accordance with the kit instructions. The TUNEL-positive neurons emit green fluorescence. DAPI (1:30, C0065, Solarbio, Beijing, China) was used for nuclear staining. The TUNEL-positive neurons were observed and photographed under the fluorescence microscope(BX51) and then quantified using the Image-Pro Plus software. Three non-overlapping fields of view were selected randomly for counting the TUNEL-positive neurons.

### Dichlorodihydrofluorescein diacetate (DCFH-DA) staining

DCFH-DA staining is a commonly used method for determining the levels of reactive oxygen species (ROS). In this staining, the neurons were first cultured under relevant conditions for 48 h and then subjected to ROS detection using the ROS assay kit (S0033, Beyotime, Shanghai, China) strictly in accordance with the kit instructions. The ROS fluorescence intensity was observed under the fluorescence microscope(BX51) and quantified using the Image-Pro Plus software.

### Adenosine triphosphate (ATP) content detection experiment

Neurons cultured under relevant conditions for 48 h were collected, and a total of 1 × 10^6^ neurons were lysed in the lysis buffer (P0013B, Beyotime, Shanghai, China). The supernatant was obtained through centrifugation at 12,000 rpm for 10 min. The ATP level in the supernatant was determined using the ATP assay kit (S0026, Beyotime, Shanghai, China) in accordance with the kit instructions.

### Mitochondrial membrane potential measurement

JC-1 staining combined with flow cytometry was employed to determine the mitochondrial membrane potential of mouse hippocampal neurons. In the normal state of mitochondrial membrane potential, JC-1 enters the mitochondrial membrane to form aggregates that are capable of emitting red fluorescence. On the other hand, when the mitochondrial membrane potential decreases, these aggregates break down into monomeric forms capable of emitting green fluorescence. In the present study, the mitochondrial membrane potential assay kit (C2006, Beyotime, Shanghai, China) was used according to the kit instructions for determining the mitochondrial membrane potential of neurons. In brief, neurons were treated with JC-1 (10 µg/mL) for 45 min in the dark and then analyzed using the BD Accuri C6 flow cytometer (BD Bioscience, San Jose, CA, USA).

### The mRFP-eGFP-LC3 assay

The mRFP-GFP-LC3 dual-fluorescent autophagy indicator system is a common tool used in autophagy evaluation. Mouse hippocampal neurons were cultured under relevant conditions for 48 h and then treated with GFP-LC3 and mRFP-EGFP-LC3 lentivirus (GT-AP-V103, Xingtuo Biomedical Technology, Shanghai, China) according to manufacturer’s instructions. The formation of autophagosomes was observed by counting the LC3puncta under a fluorescence microscope (BX51).

### Isolation of mitochondria from mouse hippocampus tissues and mouse hippocampal neurons

Mitochondria were isolated from mouse hippocampus tissues and mouse hippocampal neurons using the Mitochondrial Isolation Kit (C3601, Beyotime, Shanghai, China) in accordance with the directions. Briefly, after 48 h of cell culture, mousehippocampal neurons were harvested and suspended in 200 µL of mitochondrial isolate reagent for homogenization. In order to retrieve the mitochondria from the mouse hippocampus tissues, these tissues were excised into pieces and then homogenized in 1 mL of the mitochondria isolate reagent. After homogenization, the samples were centrifuged at 10,000 rpm for 10 min at 4 °C. Thereafter, the supernatant was collected and centrifuged at 17,000 rpm for 20 min at 4 °C. The mitochondria precipitated at the bottom of the centrifuge tube, from where they were collected and used for the subsequent WB analysis.

### Real-time quantitative reverse transcription-polymerase chain reaction (qRT-PCR)

Total RNA was extracted from the hippocampus of mice and the isolated mouse hippocampal neurons using the Total RNA Extraction Kit (R1200, Solarbio, Beijing, China). To synthesize the cDNA template, a Commercial Reverse Transcription Kit (fsq-101, Songlei Biotechnology, Shanghai, China) was employed, following the provided instructions. The qRT-PCR assay was performed using the Thermal Cycler Dice^®^ Real Time System II (TB800, TaKaRa, Japan) under the following conditions: initial denaturation at 95 °C for 30 s, followed by 40 cycles of denaturation at 95 °C for 5 s, annealing and extension at 60 °C for 30 s. The specific primers used were as follows: NTRK1 (forward: 5′-CCAGGGTGATCTCAAAGCATCTAAC-3′; reverse: 5′-CCATCTAGCGAGGCAGGAACA-3′) and Glyceraldehyde-3-phosphate dehydrogenase (GAPDH) (forward: 5′-GATGGGCGTGAACCATGAGA-3′; reverse: 5′-AGTGGTCATGGATGACTTTGGCTA-3′). The relative expression of NTRK1 mRNA was determined using the 2-ΔΔCt method and normalized to GAPDH.

### Western blotting (WB)

The total protein was extracted from sheared mouse hippocampal tissues or mouse hippocampal neurons (collected after 48 h of culture) using the radio-immunoprecipitation assay (RIPA) buffer (R0020, Solarbio, Beijing, China) according to the manufacturer’s instructions. The specific extraction of mitochondrial proteins was performed using the Mitochondrial Protein Extraction Kit (KGP8100, Kaiji Biotechnology, Jiangsu, China)in accordance with the instructions. A volume of 30 µL of the protein sample was subjected to sodium dodecyl sulfate-polyacrylamide gel electrophoresis (SDS-PAGE), and the separated proteins were transferred to polyvinylidene fluoride (PVDF) membranes. The PVDF membranes had been previously submerged in 5% skimmed milk (D8340, Solarbio, Beijing, China) for 1 h at room temperature to block the proteins. After the transfer of the proteins, the PVDF membranes were incubated with primary antibodies at 4 °C for 12 h and then with the goat anti-rabbit secondary antibody (1:2000, 4030–05, AmyJet Scientific, Wuhan, China) for 2 h at room temperature. The primary antibodies used were rabbit anti-NTRK1 (1:1000, PAB17918, AmyJet Scientific, Wuhan, China), rabbit anti-AMPK (1:1000, A-ABV10093, AmyJet Scientific, Wuhan, China), rabbit anti-p-AMPK (1:1000, YT639, Biolab Technology, Beijing, China), rabbit anti-ULK1 (1:1000, ABP56727, AmyJet Scientific, Wuhan, China), rabbit anti-p-ULK1 (1:1000, YB-19599, Yubo Biotechnology, Shanghai, China), rabbit anti-FUNDC1 (1:1000, PAB23345, AmyJet Scientific, Wuhan, China), rabbit anti-p-FUNDC1 (1:1000, AF0001, Affinity Biosciences, USA), rabbit anti-LC3 (1:1000, A-ABV11461, AmyJet Scientific, Wuhan, China), rabbit anti-P62 (1:1000, VB2368–100, AmyJet Scientific, Wuhan, China), rabbit anti-Parkin (1:1000, A-AJ1587a, AmyJet Scientific, Wuhan, China), rabbit anti-PINK1 (1:1000, PAB4484, AmyJet Scientific, Wuhan, China), rabbit anti-GAPDH(1:1000, A-AJ1089a, AmyJet Scientific, Wuhan, China), and rabbit anti-COXIV (1:1000, SPC-1320D, AmyJet Scientific, Wuhan, China). After the above incubations, the enhanced chemiluminescence reagent (SW2020, Solarbio, Beijing, China) was added to the PVDF membranes for the development of protein blots. Finally, the protein level was then qualified using the Image-Pro Plus software (Media Cybernetics, Bethesda, MD, USA). GAPDH was used as the control for total proteins in the tissues or neurons, while COXIV was used as the reference for mitochondrial proteins. All of the original western blots were shown in Supplemental Material.

### Statistical analysis

The result data from three independent replicate experiments were processed into mean ± standard deviation. Pair-wise comparison between any two groups was conducted using a two-tailed paired Student’s *t*-test. The analysis of variance together with posthoc Tukey’s test was performed for comparisons among three groups. Data met the normal distribution. *P* < 0.05 indicated a statistically significant difference.

## Supplementary information

Supplementary matrerial 1

## Data Availability

The datasets used and/or analyzed during the current study are available from the corresponding author on reasonable request.
