# Supplementary material for: NTRK1 knockdown induces mouse cognitive impairment and hippocampal neuronal damage through mitophagy suppression via inactivating the AMPK/ULK1/FUNDC1 pathway
Source: Cell Death Discov. 2023 Oct 31;9:404. doi: 10.1038/s41420-023-01685-7 (PMC10618268; doi:10.1038/s41420-023-01685-7)
Supplement: Supplementary file 1 — Supplementary matrerial 1 [file 41420_2023_1685_MOESM1_ESM.pdf]

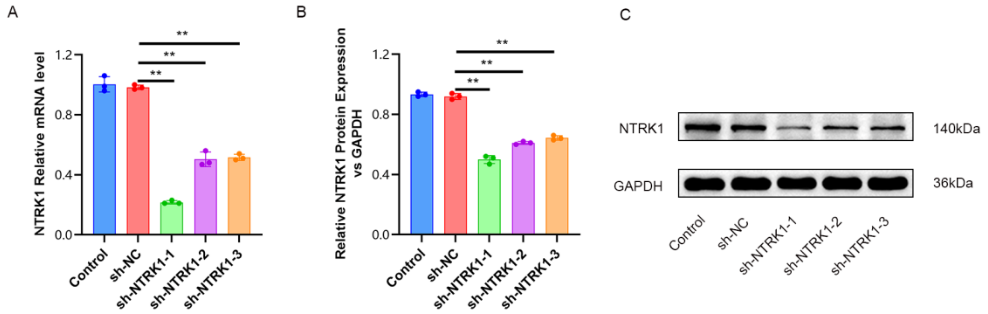

Figure S1. The research of the off-target effect of NTRK1 shRNA.

(A, B and C) The qRT-PCR and WB showed thatNTRK1 shRNA-1 had the highest transfection efficiency in the hippocampus of the mice. n = 3. ** *P*< 0.01.

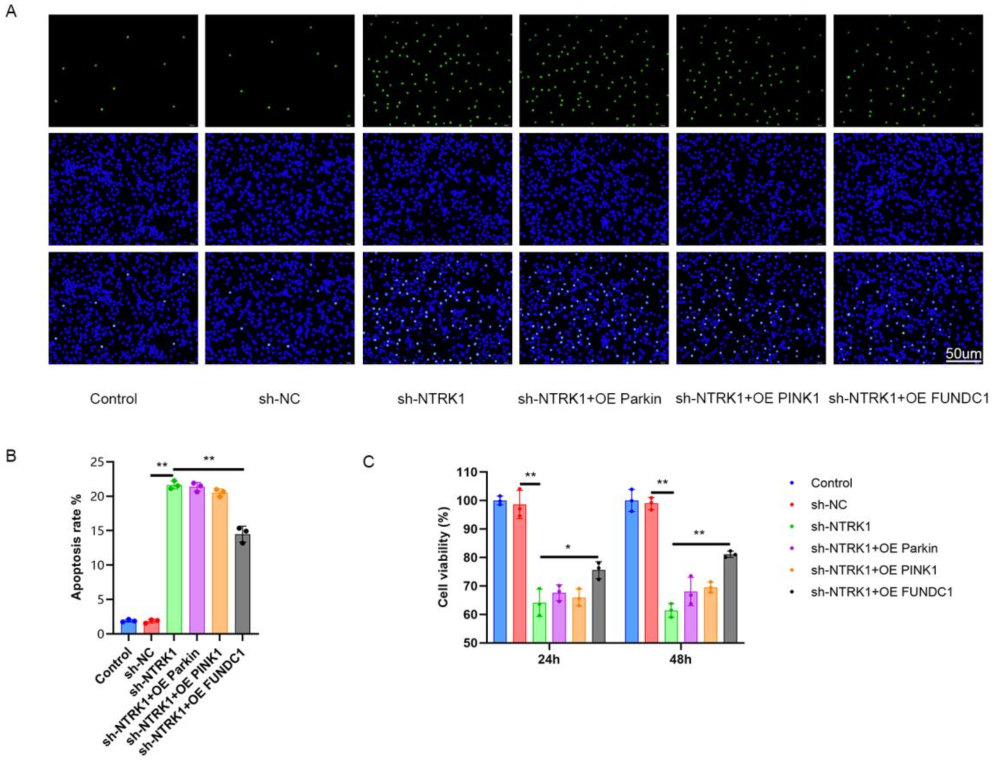

Figure S2. NTRK1 knockdown might suppress the mitophagy in mouse neurons by mediating FUNDC1.

(A and B) TUNEL assay illustrated that NTRK1 knockdown might be through mediating FUNDC1 (rather than Parkin and PINK1) to suppress mitophagy to induce the apoptosis of mouse neurons. n = 3. (C) CCK-8 assay indicated that NTRK1 knockdown might suppress mitophagy of mouse neurons to attenuate its viability by mediating FUNDC1 (rather than Parkin and PINK1). n = 3.* $P < 0.05$. ** $P < 0.01$.

Supplementary Materials 1: Uncropped Western Blot Images

Legend: All the original images of western blotting (WB) used in the figures of the main text figures. The following images correspond to WB pictures with corresponding panel numbers in the main text figures. The text on the right of the image corresponds to the name of the corresponding primary antibody.

Figure 1A:

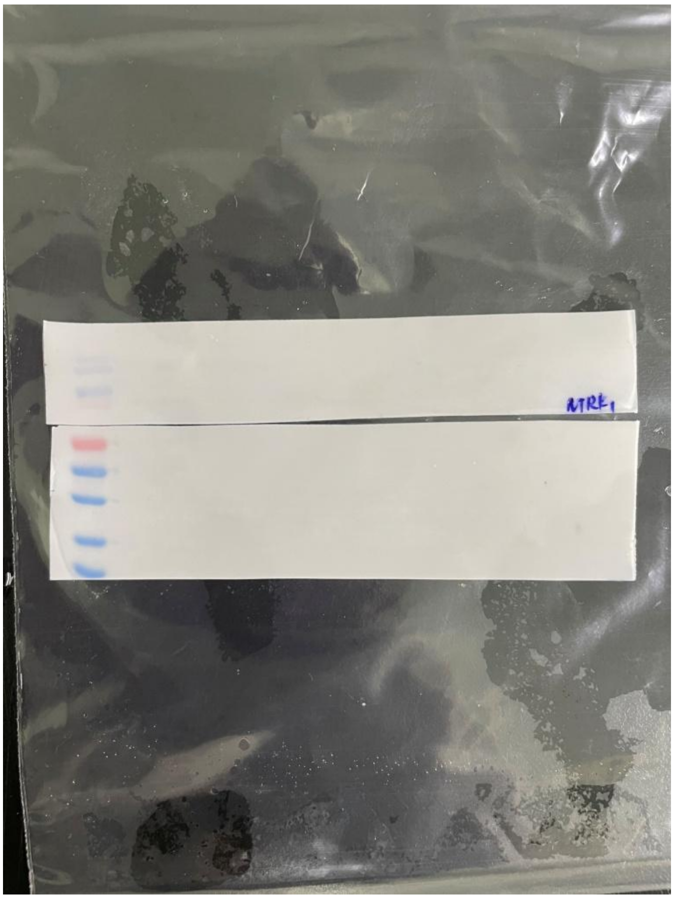

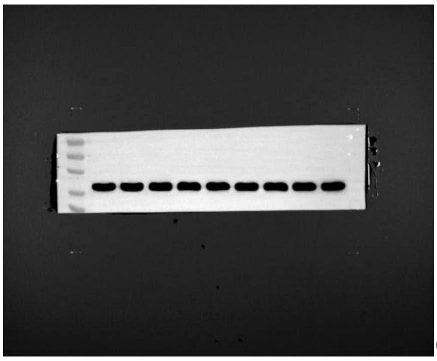
GAPDH
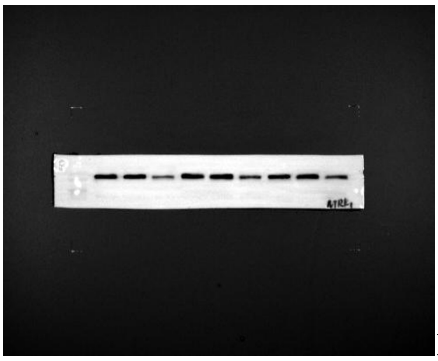
NTRK1

Figure 2I:

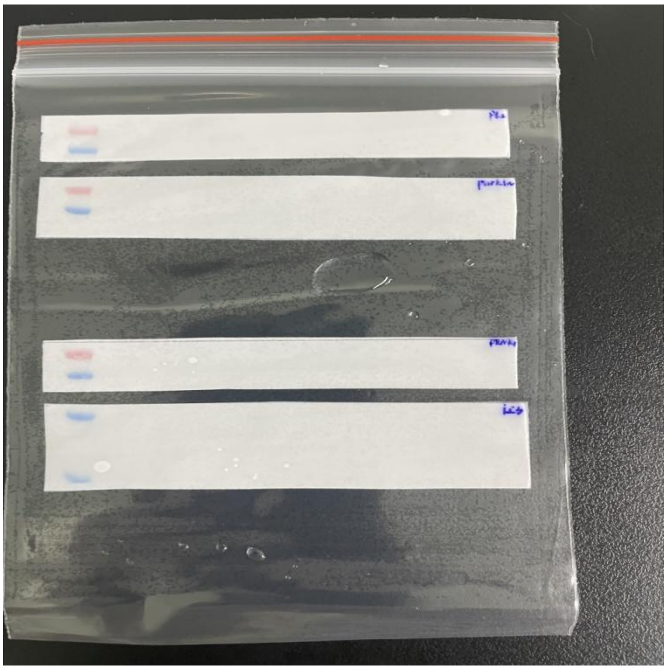

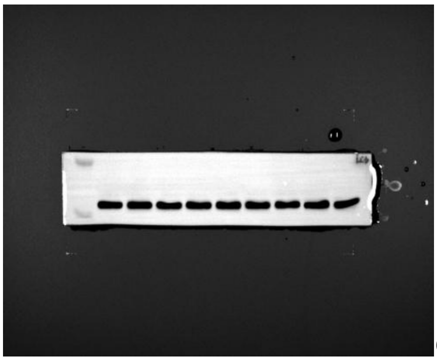

COXIV

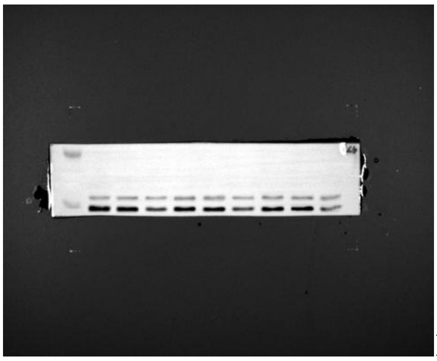

LC3

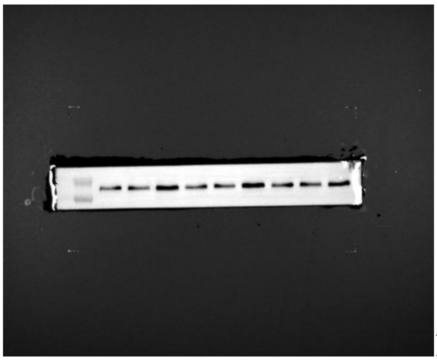

P62

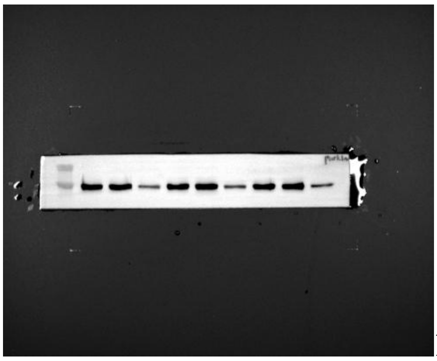

Parkin

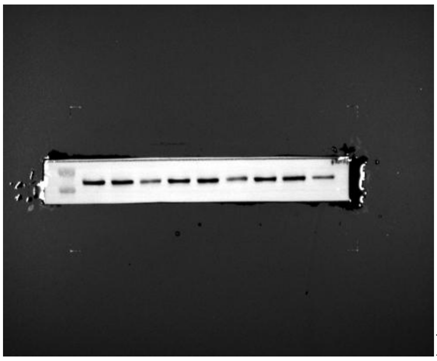
PINK1

Figure 2K:

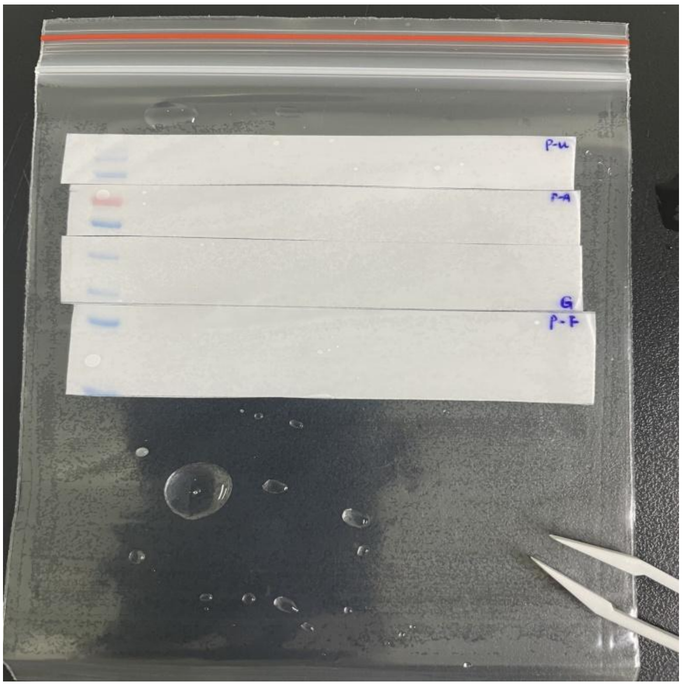

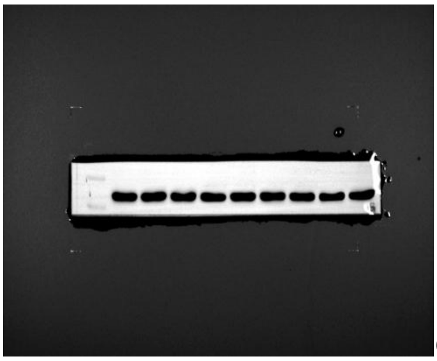
GAPDH

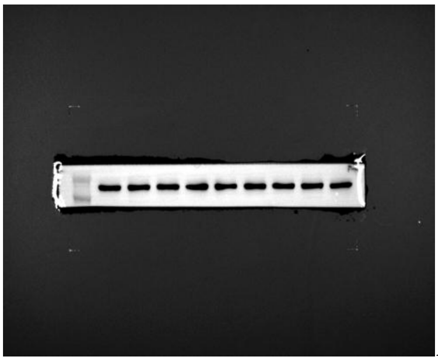
AMPK

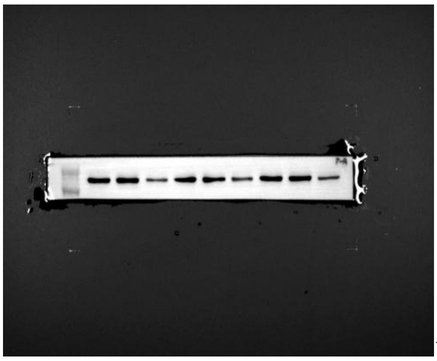
p-AMPK

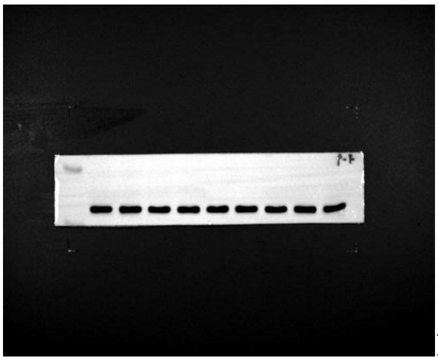
FUNDC1

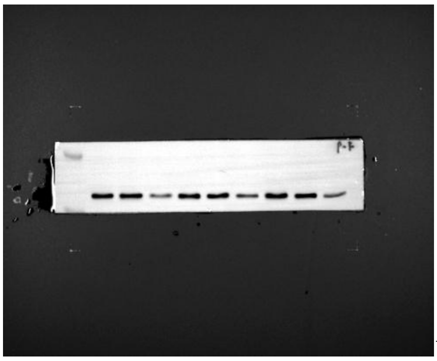
p-FUNDC1

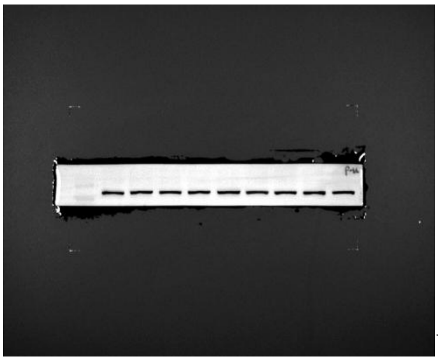
ULK1

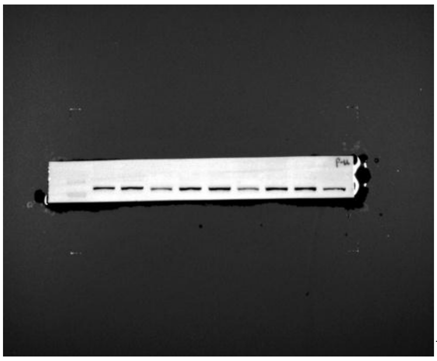
p-ULK1

Figure 5A:

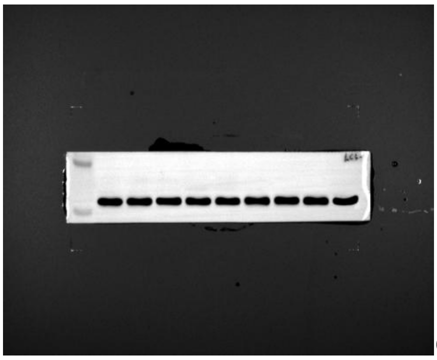
COXIV

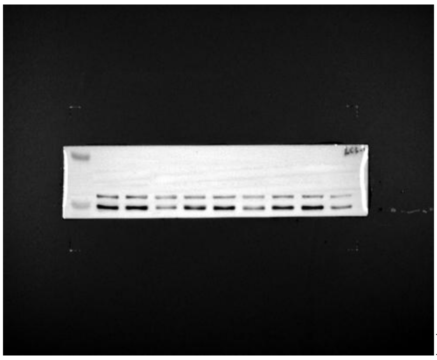
LC3

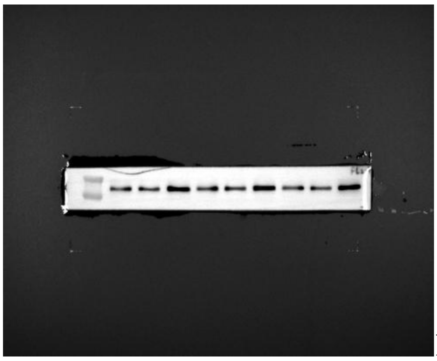
P62

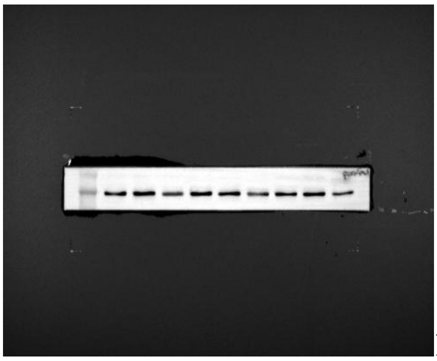
Parkin

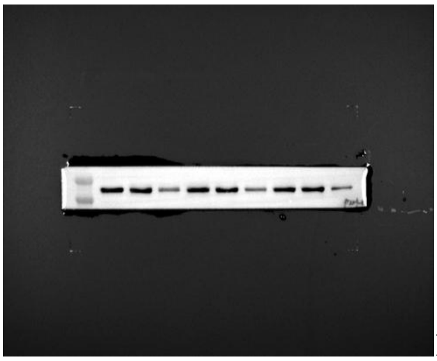
PINK1

Figure 6A:

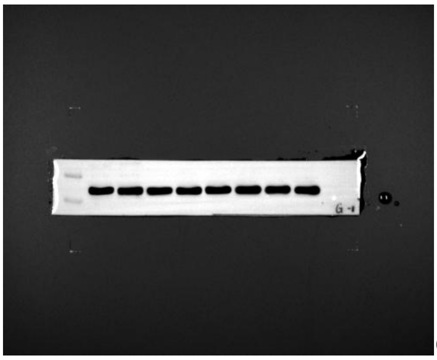
GAPDH

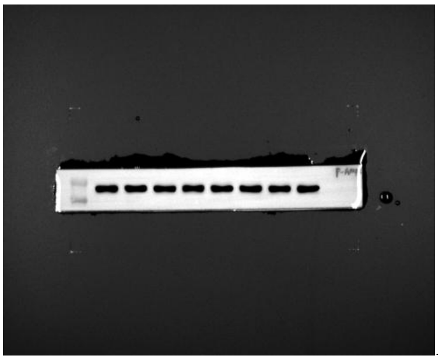
AMPK

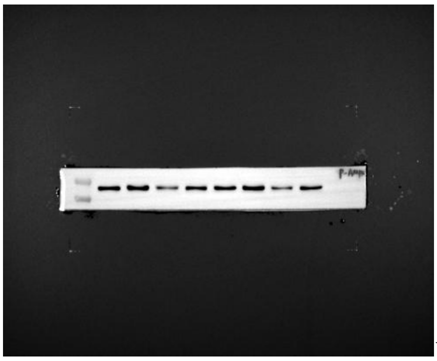
p-AMPK

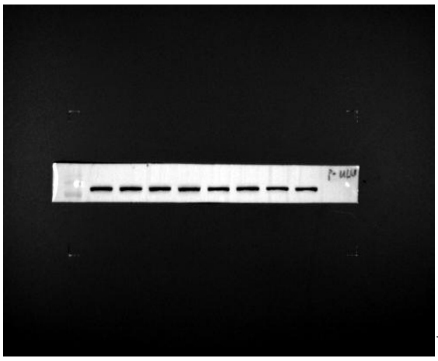
ULK1

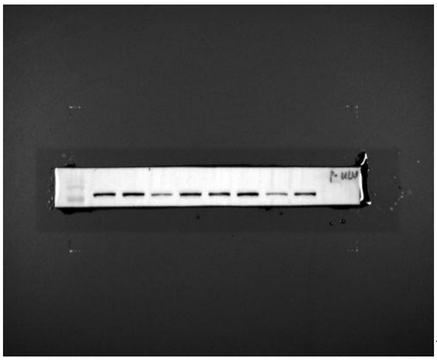
p-ULK1

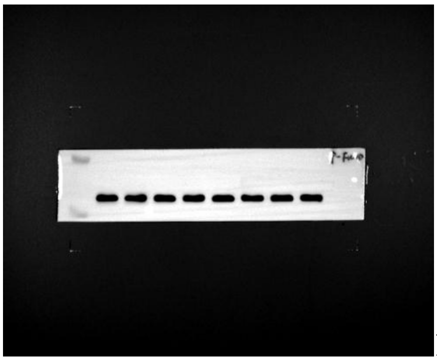
FUNDC1

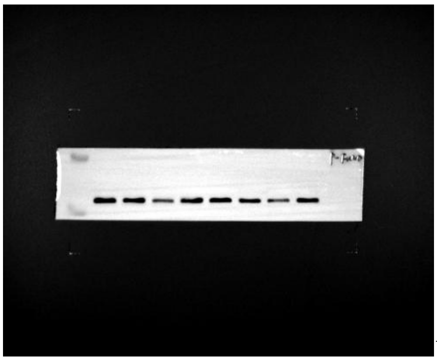
p-FUNDC1

Figure 6C:

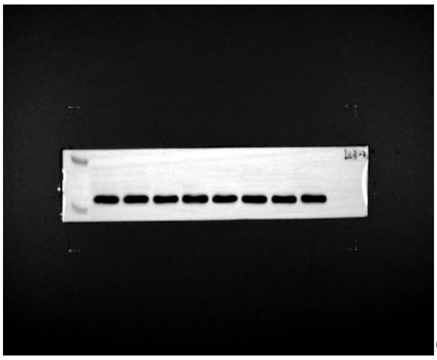
COXIV

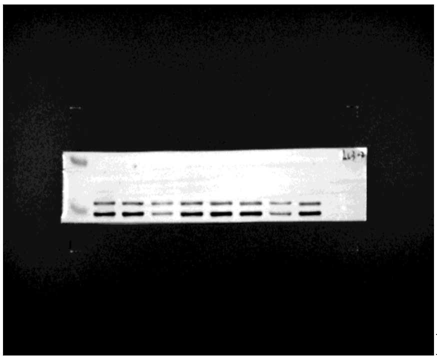
LC3

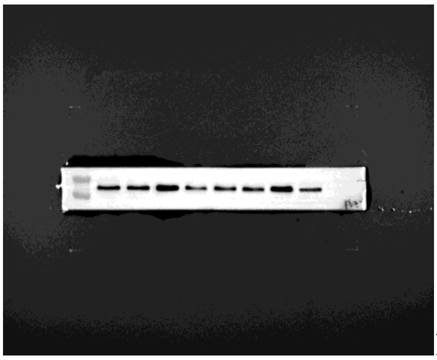
P62

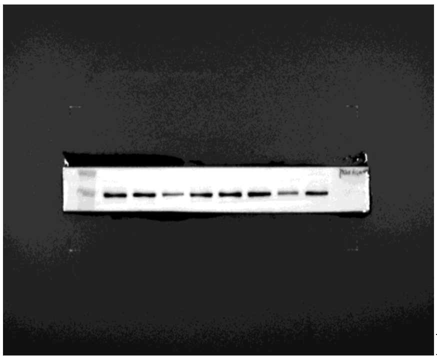
Parkin

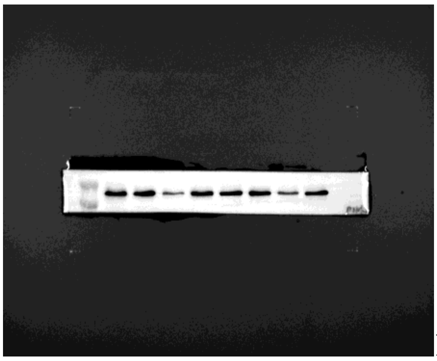

PINK1

Figure 7H:

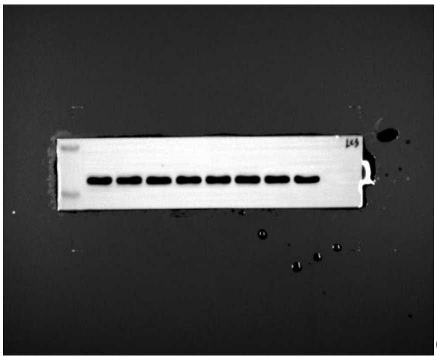

COXIV

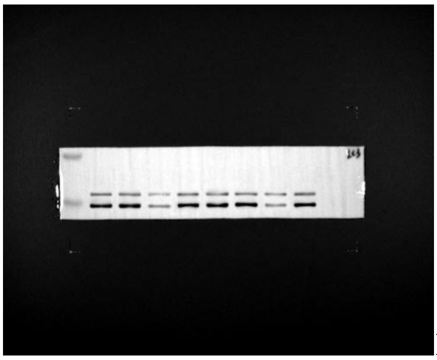

LC3

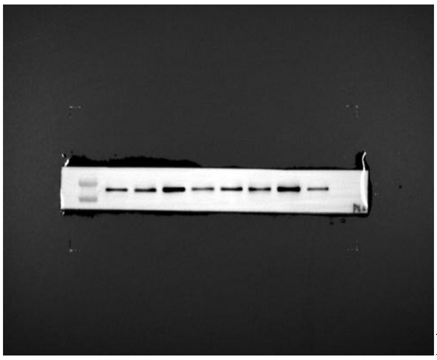

P62

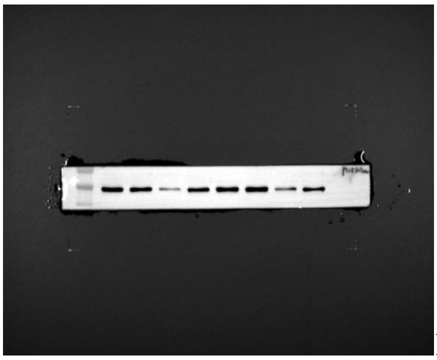

Parkin

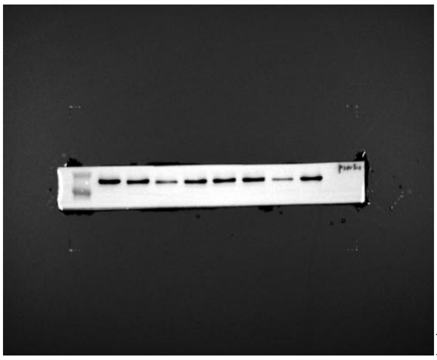

PINK1

Figure 8F:

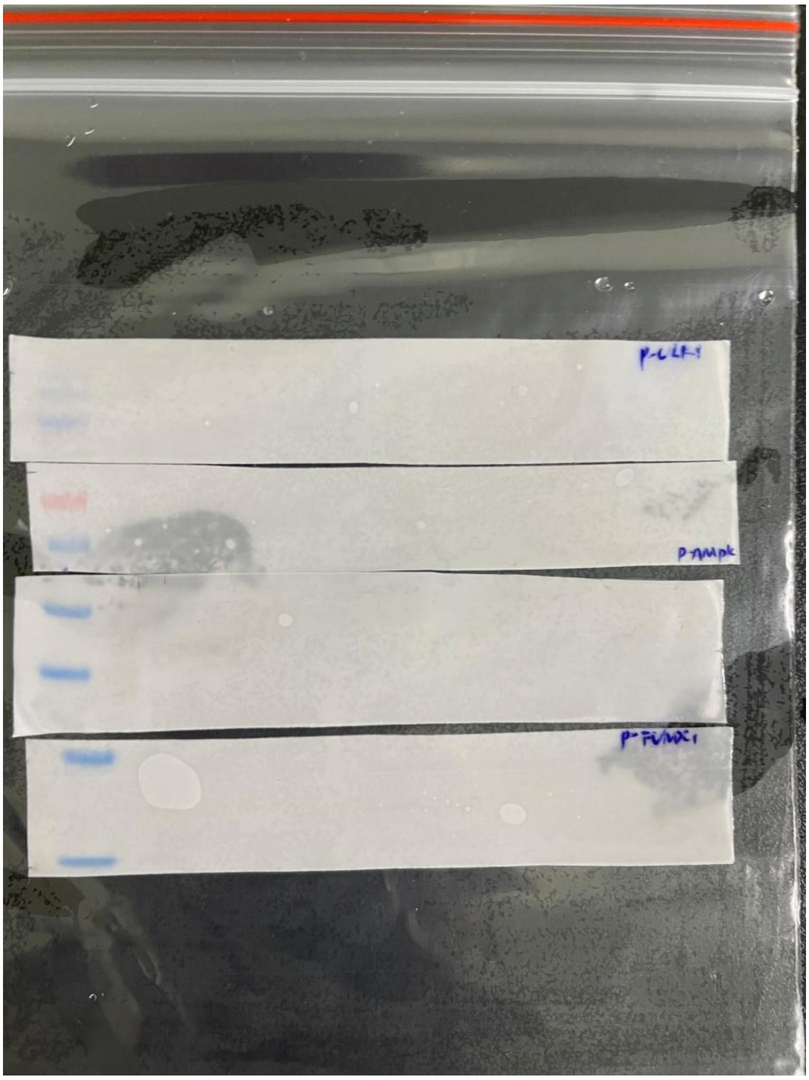

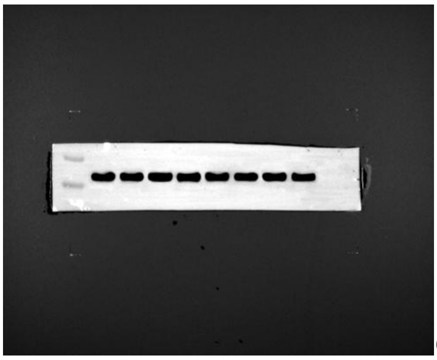
GAPDH

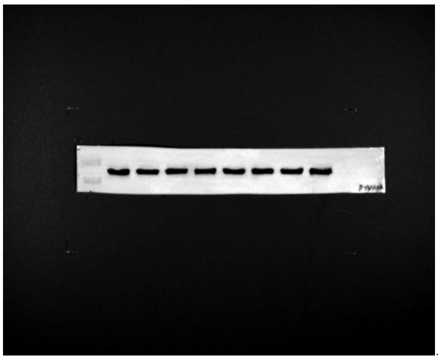
AMPK

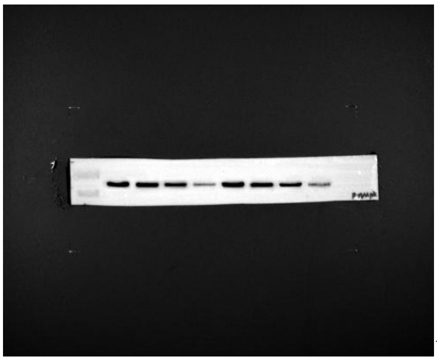
p-AMPK

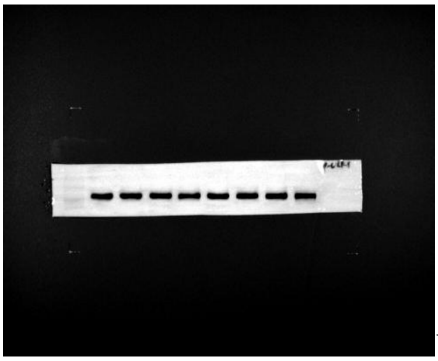
ULK1

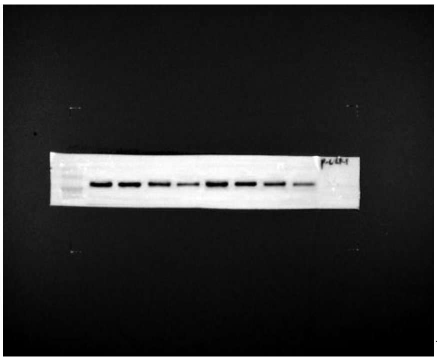
p-ULK1

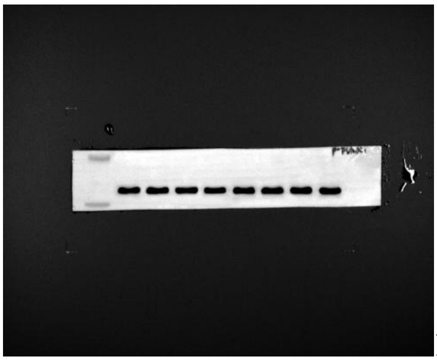
FUNDC1

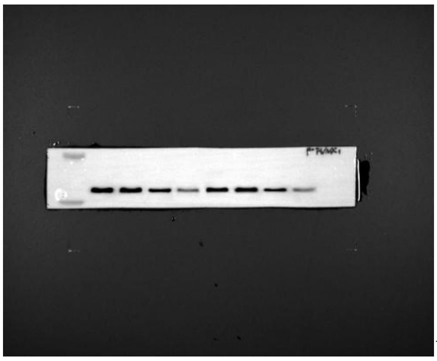
p-FUNDC1
